# Dynamics of genome architecture and chromatin function during human B cell differentiation and neoplastic transformation

**DOI:** 10.1038/s41467-020-20849-y

**Published:** 2021-01-28

**Authors:** Roser Vilarrasa-Blasi, Paula Soler-Vila, Núria Verdaguer-Dot, Núria Russiñol, Marco Di Stefano, Vicente Chapaprieta, Guillem Clot, Irene Farabella, Pol Cuscó, Marta Kulis, Xabier Agirre, Felipe Prosper, Renée Beekman, Silvia Beà, Dolors Colomer, Hendrik G. Stunnenberg, Ivo Gut, Elias Campo, Marc A. Marti-Renom, José Ignacio Martin-Subero

**Affiliations:** 1 Institut d’Investigacions Biomèdiques August Pi i Sunyer (IDIBAPS), Barcelona, Spain; 2 Departament de Fonaments Clínics, Facultat de Medicina, Universitat de Barcelona, Barcelona, Spain; 3 CNAG-CRG, Centre for Genomic Regulation (CRG), Barcelona Institute of Science and Technology (BIST), Barcelona, Spain; 4 Centro de Investigación Biomédica en Red de Cáncer (CIBERONC), Madrid, Spain; 5 Gastrointestinal and Endocrine Tumors Group, Vall d’Hebron Institute of Oncology (VHIO), Barcelona, Spain; 6 Área de Oncología, Centro de Investigación Médica Aplicada (CIMA), Instituto de Investigación Sanitaria de Navarra (IdiSNA), Universidad de Navarra, Pamplona, Spain; 7 Departamento de Hematología, Clínica Universidad de Navarra, Universidad de Navarra, Pamplona, Spain; 8 Hematopathology Section, Hospital Clinic of Barcelona, Barcelona, Spain; 9 Molecular Biology, NCMLS, FNWI, Radboud University, Nijmegen, The Netherlands; 10 Universitat Pompeu Fabra (UPF), Barcelona, Spain; 11 Institució Catalana de Recerca i Estudis Avançats (ICREA), Barcelona, Spain; 12 Centre for Genomic Regulation (CRG), Barcelona Institute of Science and Technology (BIST), Barcelona, Spain

**Keywords:** Haematological cancer, Differentiation, Gene regulation, B cells, Chromatin structure

## Abstract

To investigate the three-dimensional (3D) genome architecture across normal B cell differentiation and in neoplastic cells from different subtypes of chronic lymphocytic leukemia and mantle cell lymphoma patients, here we integrate in situ Hi-C and nine additional omics layers. Beyond conventional active (A) and inactive (B) compartments, we uncover a highly-dynamic intermediate compartment enriched in poised and polycomb-repressed chromatin. During B cell development, 28% of the compartments change, mostly involving a widespread chromatin activation from naive to germinal center B cells and a reversal to the naive state upon further maturation into memory B cells. B cell neoplasms are characterized by both entity and subtype-specific alterations in 3D genome organization, including large chromatin blocks spanning key disease-specific genes. This study indicates that 3D genome interactions are extensively modulated during normal B cell differentiation and that the genome of B cell neoplasias acquires a tumor-specific 3D genome architecture.

## Abstract (web summary)

The dynamics of genome architecture during human cell differentiation and upon neoplastic transformation remain poorly characterized. Here, the authors integrate in situ Hi-C and nine additional omic layers to characterize the dynamic changes in 3D genome architecture during normal B cell differentiation and in neoplastic cells from chronic lymphocytic leukemia and mantle cell lymphoma patients.

## Introduction

Over the last decades, our understanding of higher-order chromosome organization in the eukaryotic interphase nucleus and its regulation of cell state, function, specification, and fate has profoundly increased^1,2^.

Chromatin conformation capture techniques have been used to elucidate the genome compartmentalization^3,4^. It is widely accepted that the genome is segregated into two large compartments, named A-type and B-type^5^, which undergo widespread remodeling during cell differentiation^2,6–9^. These compartments have been associated with different GC content, DNAseI hypersensitivity, gene density, gene expression, replication time, and chromatin marks^5,10^. Alternative subdivisions of genome compartmentalization have been proposed, including three compartments^11^ or even six compartment subtypes with distinct genomic and epigenomic features^12^. All of these studies highlight the role of three-dimensional (3D) genome organization in the regulatory decisions associated with cell fate. However, the majority of these studies have been performed using cell lines, animal models, or cultured human cells^7,8,13–15^, and although few analyze sorted cells from healthy human individuals^16–18^, there is limited information regarding 3D genome dynamics across the differentiation program of a single human cell lineage^16^.

Normal human B cell differentiation is an ideal model to study the dynamic 3D chromatin conformation during cell maturation, as these cells show different transcriptional features and biological behaviors, and can be accurately isolated due to their distinct surface phenotypes^19,20^. Moreover, how the 3D genome of B cells is modulated upon neoplastic transformation using primary samples from patients is also widely unknown^21^. In this context, several types of neoplasms can originate from B cells at distinct differentiation stages^22^. Out of them, chronic lymphocytic leukemia (CLL) and mantle cell lymphoma (MCL) are derived from mature B cells and show a broad spectrum of partially overlapping biological features and clinical behaviors^23^. Both diseases can be categorized according to the mutational status of the immunoglobulin heavy chain variable region (IGHV), a feature that seems to be related to the maturation stage of the cellular origin^24^. CLL cases lacking IGHV somatic hypermutation are derived from germinal center-independent B cells whereas CLL with mutated IGHV derives from germinal center-experienced B cells^25^. In CLL, this variable is strongly associated with the clinical features of the patients, with mutated IGHV (mCLL) cases correlating with good prognosis and those lacking IGHV mutation (uCLL) with poorer clinical outcome^25^. In MCL, although two groups based on the IGHV mutational status can be recognized and partially correlate with clinical behavior, other markers such as expression of the *SOX11* oncogene are used to classify cases into clinically aggressive conventional MCL (cMCL) and clinically indolent non-nodal leukemic MCL (nnMCL)^23,26–28^.

From an epigenomic perspective, previous reports have identified that B cell maturation and neoplastic transformation to CLL or MCL entail extensive modulation of the DNA methylome and histone modifications^29–34^. However, whether such epigenetic changes are also linked to modulation of the higher-order chromosome organization is yet unknown^35^.

Here, to decipher the 3D genome architecture of normal and neoplastic B cells, we generated in situ high-throughput chromosome conformation capture (Hi-C) maps of cell subpopulations spanning the B cell maturation program as well as of neoplastic cells from MCL and CLL patients. Next, we mined the data together with whole-genome maps of six different histone modifications, chromatin accessibility, DNA methylation, and gene expression obtained from the same human cell subpopulations and patient samples. This multi-omics approach not only allowed us to describe a widespread modulation of the chromosome organization and function during human B cell maturation and neoplastic transformation but also provides a unique dataset that shall represent a valuable asset for future studies in the field of cell differentiation and immunological cancer.

## Results

### Multi-omics analysis during human B cell differentiation

**Fig. 1:**
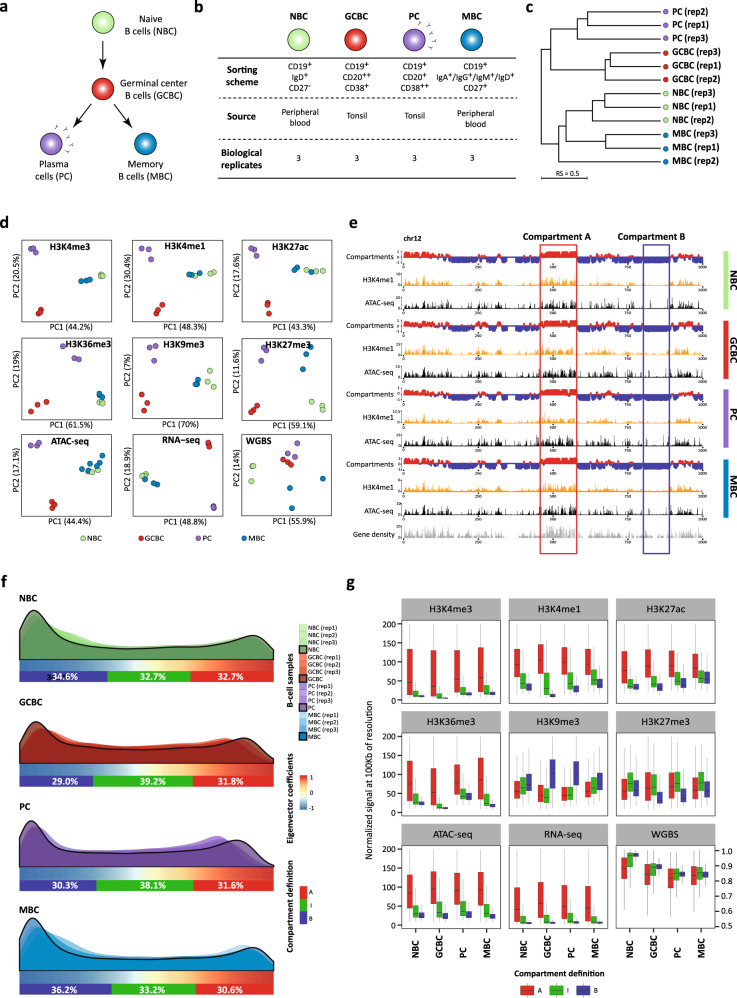
Multi-omics view of B cell differentiation and identification of an intermediate compartment. **a** Schematic overview of mature B cell differentiation showing the four B cell subpopulations considered in this study. **b** Table of sample description together with the number of in situ Hi-C replicates analyzed by each B cell subpopulations. **c** Dendrogram of the reproducibility scores of B cell subpopulation replicates for normalized Hi-C contact maps at 100 kb resolution. **d** Unsupervised principal component analysis (PCA) for nine omics layers: ChIP-seq of six histone marks (H3K4me3 *n* = 46,184 genomic regions, H3K4me1 *n* = 44,201 genomic regions, H3K27ac *n* = 72,222 genomic regions, H3K36me3 *n* = 25,945 genomic regions, H3K9me3 *n* = 40,704 genomic regions, and H3K27me3 *n* = 20,994 genomic regions), chromatin accessibility measured by ATAC-seq (*n* = 99,327 genomic regions), DNA methylation measured by WGBS (*n* = 15,089,887 CpGs) and gene expression measured by RNA-seq (*n* = 57,376 transcripts). **e** Example of chromosome 12 (chr12) comparing the eigenvector profile, H3K4me1 ChIP-seq signal, chromatin accessibility (ATAC-seq), and gene density. The red and blue rectangles highlight the features of A and B compartments, respectively. The scale indicates the genomic position in kilobase pairs (kb). **f** Distribution of the first eigenvector of each B cell subpopulation (three replicates and merge). The relative abundance of A-type, B-type, and intermediate (I)-type compartments per merged B cell subpopulations are indicated below each distribution. Compartment definition based on eigenvalue thresholds: A-type, +1.00 to +0.43; I-type, +0.43 to −0.63; B-type, −0.63 to −1.00. **g** Boxplots showing the association of the three compartments (A-type, I-type, and B-type) with each of the nine additional omics layers under study. The median signal of each layer was computed for each B cell subpopulation. For the boxplots, centerline, box limits, and whiskers represent the median, 25th, and 75th percentiles and 1.5× interquartile range, respectively. Sample sizes were NBC (naive B cells), GCBC (germinal center B cells), MBC (memory B cells), and PC (plasma cells): *n* = 3 biologically independent samples (all nine layers with the exception of ATAC-seq for MBC which *n* = 6 biologically independent samples were used).

We used in situ Hi-C to generate genome-wide chromosome conformation maps of normal human B cells across their maturation program. These included three biological replicates each of naive B cells (NBC), germinal center B cells (GCBC), memory B cells (MBC), and plasma cells (PC) (Fig. 1a, b and Supplementary Data 1). From the same B cell subpopulations, we analyzed nine additional omics layers generated as part of the BLUEPRINT consortium^29,36^. Specifically, we obtained data for chromatin immunoprecipitation with massively parallel sequencing (ChIP-seq) of six histone modifications with non-overlapping functions (H3K4me3, H3K4me1, H3K27ac, H3K36me3, H3K9me3, H3K27me3), transposase-accessible chromatin with high-throughput sequencing (ATAC-seq), whole-genome bisulfite sequencing (WGBS), and gene expression (RNA-seq).

We initially explored the intra- and inter-subpopulation variability and observed that the Hi-C replicas were concordant, as quantified measuring and clustering the reproducibility score (RS)^37^ (Fig. 1c and Supplementary Fig. 1a). Furthermore, the comparison of samples suggests that the overall genome architecture of NBC is more similar to MBC and clearly different from GCBC and PC, which belong to a different cluster (Fig. 1c). This finding was also reflected in the first component of the principal component analysis (PCA) of histone modifications, chromatin accessibility, and gene expression (Fig. 1d). In contrast to other omics marks, the first component of DNA methylation data resulted in a division of GCBC, MBC, and PC separated from the NBC. These analyses suggest fundamental differences between chromatin-based epigenetic marks, including chromosome conformation data, and DNA methylation. In fact, changes in DNA methylation linearly accumulate throughout B cell maturation^31,32^, which explains the clear differences between NBC and MBC in spite of their converging transcriptomes.

### Polycomb-associated chromatin defines an intermediate and moldable 3D genome compartment

**Fig. 2:**
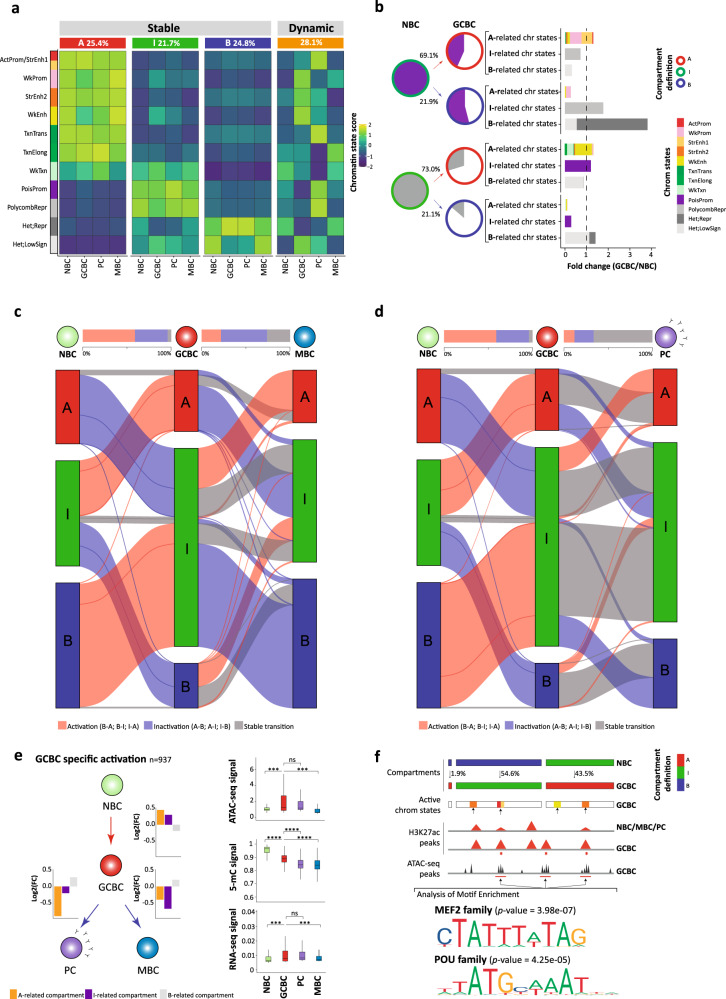
Chromatin dynamics across B cell differentiation. **a** Functional association of the stable (segmented into A-type, I-type, and B-type) and dynamic compartments during B cell maturation using 11 chromatin states. The percentage of each stable or dynamic compartment is indicated for all B cell subpopulations. **b** Intermediate compartment dynamics. The filled area of the pie charts represents the percentage of the poised promoter (top, violet) or polycomb-repressed (bottom, gray) states within the I-type compartment in NBC which shifts to A-type and B-type compartments in GCBC. The pie charts under GCBC represent the fraction that maintains the previous chromatin state (colored as previously defined) or changes chromatin states (not colored). The outline color of the pie charts represents the compartment type. Bar graphs represent the fold change between GCBC and NBC of each of the three groups of chromatin states (arranged by their relationship to the A-type, I-type, and B-type compartments). **c**, **d** Alluvial diagrams showing the compartment dynamics in the two branches of mature B cell differentiation: NBC-GCBC-MBC (**c**) and NBC-GCBC-PC (**d**). Compartment changes are represented in red if they show activation (as B-type to A-type, B-type to I-type, and I-type to A-type), in blue, in case of inactivation (as A-type to B-type, A-type to I-type, and I-type to B-type) or in gray for non-changed compartments. At the top, the bar plots between B cell subpopulations represent the total percentage of regions changing to active or inactive, and regions that conserve its previous compartment definition. **e** Multi-omics characterization of the 937 regions (at 100 kb resolution) gaining activity exclusively in GCBC. *Left*: Scheme of B cell differentiation and chromatin state dynamics, in which the bar plots indicate the log2 fold change of active, intermediate, or inactive-related chromatin state groups. *Right*: Boxplots of chromatin accessibility (ATAC-seq signal), DNA methylation (5-mC signal), and gene expression (RNA-seq signal) per B cell subpopulations. The median signal of each layer was computed for NBC, GCBC, PC, and MBC: *n* = 3 biologically independent samples (all nine layers with the exception of ATAC-seq for MBC which *n* = 6 biologically independent samples were used). For the boxplots, centerline, box limits, and whiskers represent the median, 25th, and 75th percentiles and 1.5× interquartile range, respectively. *P* values were calculated using the Wilcoxon rank-sum test (two-sided); ns nonsignificant, ***p value < 0.001, ****p value < 0.0001. **f** Enrichment analysis of transcription factor (TF) binding motifs. *Top*: Schematic representation of the analytic strategy. *Bottom*: Binding motifs of MEF2 and POU TF families in active and accessible loci in the GCBC specific regions gaining activity (*n* = 171 independent genomic loci) vs. the background (*n* = 268 independent genomic loci). *P* values were calculated using the Wilcoxon rank-sum test (one-sided). ActProm-StrEnh1 active promoter-strong enhancer 1, WkProm weak promoter, StrEnh2 strong enhancer 2, WkEnh weak enhancer, TxnTrans transcription transition, TxnElong transcription elongation, WkTxn weak transcription were A-type compartment-related states. PoisProm poised promoter, PolycombRepr polycomb-repressed were I-type compartment-related states. Het;Repr heterochromatin-repressed, Het;LowSign heterochromatin-low signal were B-type compartment-related states.

To study the compartmentalization of the genome during B cell differentiation, we next merged all biological replicates per B cell subpopulation resulting in interaction Hi-C maps with around 300 million valid reads each. These Hi-C interaction maps were further segmented into positive and negative eigenvalues based on the eigenvector decomposition^5,38^, and regions were assigned to the A-type (active) and B-type (inactive) compartments using the association with histone modifications (Fig. 1e and Supplementary Fig. 1b). A pairwise correlation of the first eigenvector of each B cell subpopulation showed that NBC and MBC on the one hand, and GCBC and PC on the other hand, have similar compartmentalization (Supplementary Fig. 1c), confirming previous results using the RS (Fig. 1c). Unexpectedly, the H3K27me3 histone mark, which is deposited by the polycomb repressive complex^39^, was neither correlated with positive nor with negative eigenvector coefficients (Supplementary Fig. 1b). We then speculated that, as H3K27me3 was not related to standard A-type or B-type compartments, this histone mark may be linked to a different type of chromatin compartmentalization. In this context, a visual inspection of the first eigenvector distribution revealed a positive extreme, a negative extreme, and a long intermediate valley (Fig. 1f). Indeed, applying the Bayesian Information Criterion, we observed that classification into three compartments was the best compromise between distribution fitting accuracy and a minimum number of compartments (Supplementary Fig. 1d). Subsequently, we modeled the eigenvector distribution to establish the thresholds segmenting the data into an A-type, B-type, and intermediate (I)-type compartments (Fig. 1f and Supplementary Fig. 1e). Analyzing these three compartments together with other omics layers revealed the expected association of A-type compartment with active chromatin, B-type compartment with H3K9me3, and a remarkable association between the I-type compartment and the presence of H3K27me3 (Fig. 1g). Indeed, a chromHMM-based chromatin state model specific for B cells^29,40^ revealed that the regions associated with the I-type compartment were enriched for poised-promoter and polycomb-repressed chromatin states (Fig. 2a and Supplementary Fig. 2a).

We next quantified the compartment interactions by computing the compartment score (C-score) as the ratio of intra-compartment interactions over the total chromosomal interactions per compartment (Supplementary Fig. 2b). The I-type compartment was associated with a lower C-score than the A-type and B-type compartments (Supplementary Fig. 2c). We further explored this phenomenon by dividing the I-type compartment into two blocks differentiating positive (IA) and negative (IB) eigenvector components (Supplementary Fig. 2d). The analysis showed that the I-type compartment, regardless of being IA or IB, was consistently having a lower C-score than the A-type or B-type compartments. This finding further supports the existence of the I-type compartment as an independent chromatin structure different from A-type and B-type compartments. In addition, it suggests that the I-type compartment tends to interact not only with itself but also with A-type and B-type compartments, and as such it may represent an interconnected space between the fully active and inactive compartments.

As Rao et al.^12^ proposed a genome segmentation into six subcompartments, including one enriched in polycomb-repressed regions, we next aimed at comparing the two strategies, with particular emphasis on the I-type compartment. We observed that this I-type compartment was composed of different percentages from the six subcompartments. The I-type compartment showed the highest proportion of B1, which was described as polycomb-related but also contained significant fractions of other compartments (Supplementary Fig. 2e). Similar results were obtained when these two types of segmentations were compared using published Hi-C data from the GM12878 cell line (Supplementary Fig. 2f). These results show that, although there is some overlap between I-type and B1 compartments, they appear to reflect distinct structures. However, these differences may also be influenced by the different analytical approaches used by the two compartmentalization methods. The six subcompartments clustering was based on a subset of the inter-chromosomal contact data while a more straight-forward approach including all interactions was used in this study to determine the I-type compartment.

To study the potential role of the I-type compartment during B cell differentiation, we selected poised promoters or polycomb-repressed regions within this compartment in NBC and studied how they change in both compartment and chromatin state upon differentiation into GCBC (Fig. 2b). The majority of compartment transitions affecting these chromatin states (69.1% of the poised promoter and 73.0% of polycomb-repressed) change into A-type compartment, a consistent fraction (21.9% and 21.1%) into B-type, and only a small fraction (9.0% and 5.9%) maintain their intermediate definition. This finding indicates that the regions with a most prominent I-type compartment character undergo a widespread structural modulation during NBC to GCBC differentiation step. Transitions from I-type to A-type compartment (activation events) were paired with a reduction of poised promoters (56.7% loss) and polycomb-repressed states (70.2% loss). These reductions were associated with an increase of A-related chromatin states (1.31- or 1.33-fold change coming from the poised promoter or polycomb-repressed, respectively) such as a promoter, enhancer, and transcription (Fig. 2b). Conversely, poised promoters and polycomb-repressed regions associated with I-type compartments in NBC that changed into B compartments in GCBC (inactivation events) were related to an increase of B-related chromatin states (3.81- or 1.4-fold change coming from the poised promoter or polycomb-repressed, respectively) such as heterochromatin characterized by H3K9me3 (Fig. 2b).

Altogether, an analysis of the eigenvalue distribution of Hi-C data reveals the presence of an intermediate transitional compartment with biological significance, enriched in poised and polycomb-repressed chromatin states, interconnected with A-type and B-type compartments, and amenable to rewire the pattern of interactions leading to active or inactive chromatin state transitions upon cell differentiation.

### Changes in genome compartmentalization are reversible during B cell differentiation

Mapping A, I, and B-type compartments in NBC, GCBC, MBC, and PC Hi-C maps revealed that 28.1% of the genome dynamically changes compartment during B cell differentiation (Fig. 2a and Supplementary Fig. 2a). B cell differentiation is not a linear process, NBC differentiates into GCBC, which then branch into long-lived MBC or antibody-producing PC. Thus, we studied the 3D genome compartment dynamics along these two main differentiation paths (NBC-GCBC-PC and NBC-GCBC-MBC). At each differentiation step, we classified the genome into three different dynamics: (i) compartments undergoing activation events (B-type to A-type, B-type to I-type, or I-type to A-type), (ii) compartments undergoing inactivation events (A-type to B-type, A-type to I-type, or I-type to B-type), and (iii) stable compartments (Fig. 2c, d). The NBC-GCBC-MBC differentiation path suggests that the extensive remodeling taking place from NBC to GCBC is followed by an overall reversion of the compartmentalization in MBC, achieving a profile similar to NBC (Fig. 2c). To assess the capacity of the genome to revert to a past 3D configuration, we analyzed the compartments in NBC as compared to those in PC and MBC. Indeed, we globally observed that 72.7% of the regions in MBC re-acquire the same compartment type as in NBC. This phenomenon was mostly related to compartments undergoing activation in GCBC, as 82.9% of them reverted to inactivation upon differentiation into MBC. This finding is in line with solid evidence showing that NBC and MBC, in spite of representing markedly different maturation B cell stages, are phenotypically similar^41,42^ (Fig. 1d). In the case of PC, the compartment reversibility accounted only for 30.8% of the genome (Fig. 2d). To determine whether this compartment reversibility was also accompanied by a functional change, we analyzed the chromatin state dynamics within the compartments becoming uniquely active in GCBC as compared to NBC, MBC, and PC (*n* = 937) (Supplementary Data 2). We observed that the transient compartment activation from NBC to GCBC is related to an increase of A-related chromatin states (1.36-fold change). Conversely, the subsequent 3D genome inactivation upon differentiation into PC and MBC was related to an increase in B-related chromatin states (1.21- and 1.15-fold change, respectively) (Fig. 2e left). Furthermore, those regions had a significant increase in chromatin accessibility and gene expression in GCBC as compared to NBC and MBC, but not in PC (Fig. 2e right). These findings suggest that structural 3D reversibility in MBC is accompanied by functional reversibility whereas, in these regions, PC partially maintains gene expression levels and chromatin accessibility similar to GCBC in spite of the compartment changes. In contrast to chromatin-based marks, DNA methylation was overall unrelated to a compartment or chromatin state dynamics of the B cell differentiation (Fig. 2e right).

### The 3D genome of GCBC undergoes extensive compartment activation

**Fig. 3:**
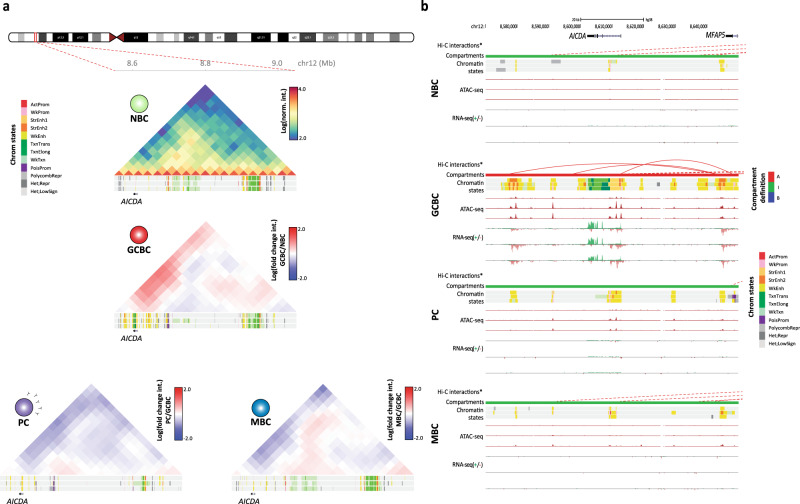
Chromatin organization at the *AICDA* locus. **a** Normalized Hi-C contact map of the domain structure surrounding the *AICDA* gene (chr12:8,598,290-8,615,591, GRCh38) in NBC. The log fold change interaction between GCBC, MBC, or PC as compared to NBC was computed. Below each interaction map, chromatin state tracks of three biological replicates per B cell subpopulation are shown. The coordinates of the represented region are chr12:8,550,000–9,050,000, GRCh38. **b** Multi-layer epigenomic characterization of the *AICDA* gene region in four B cell subpopulations. Arc diagrams indicate significant Hi-C interactions (continuous red lines involve the region of interest, while dashed red lines involve other regions of chromosome 12). Below them, compartment definition (red, compartment A-type: green, compartment I-type), chromatin states, chromatin accessibility (ATAC-seq, *y*-axis signal from 0 to 105), and gene expression (RNA-seq, *y*-axis signal from 0 to 4 for the positive strand and from 0 to −0.1 for the negative strand) are shown. Tracks of Hi-C interactions and compartment definition are based on merged replicates whereas chromatin states, chromatin accessibility, and gene expression tracks of each replicate are shown separately. The coordinates of the represented region are chr12:8,570,000–8,670,000, GRCh38.

Our analyses revealed that the NBC to GCBC transition was associated with a large structural reconfiguration of compartments involving 95.9% of all dynamic compartments. Totally, 61.5% of the changes between NBC and GCBC involved compartment activation (Fig. 2c, d). As the germinal center reaction is known to be mediated by specific transcription factors (TFs)^43,44^ and those may be involved in shaping the spatial organization of the genome^8,14,16^, we further explored the presence of TF-binding motifs in accessible chromatin in regions gaining H3K27ac within the newly activated compartments. We identified enriched motifs for several TFs, being members of the MEF2 and POU families the most significant (Fig. 2f and Supplementary Data 3), which are essential TFs involved in germinal center formation^45–48^. Furthermore, the newly activated compartments hosted 100 genes significantly upregulated in GCBC as compared to the rest of B cell subpopulations (FDR < 0.05) (Supplementary Data 4). Remarkably, among them was the activation induced cytidine deaminase (*AICDA*) gene, which is essential for class-switch recombination and somatic hypermutation in GCBC and is specifically expressed in GCBC^49^. Indeed, the *AICDA* locus was globally remodeled from an inactive state in NBC to a global chromatin activation in GCBC, which included an increase in the ratio of GCBC/NBC 3D interactions as well as increased levels of active chromatin states (that is, active promoter and enhancers as well as transcriptional elongation), open chromatin, and gene expression (Fig. 3a, b). This analysis also revealed the presence of possible upstream and downstream *AICDA*-specific enhancers that gain interactions with the gene promoter in GCBC (Fig. 3b). This multi-layer chromatin activation at the *AICDA* locus was reverted to the inactive ground state once GCBC differentiate into MBC or PC.

### B cell neoplasms undergo disease-specific 3D genome reorganization

**Fig. 4:**
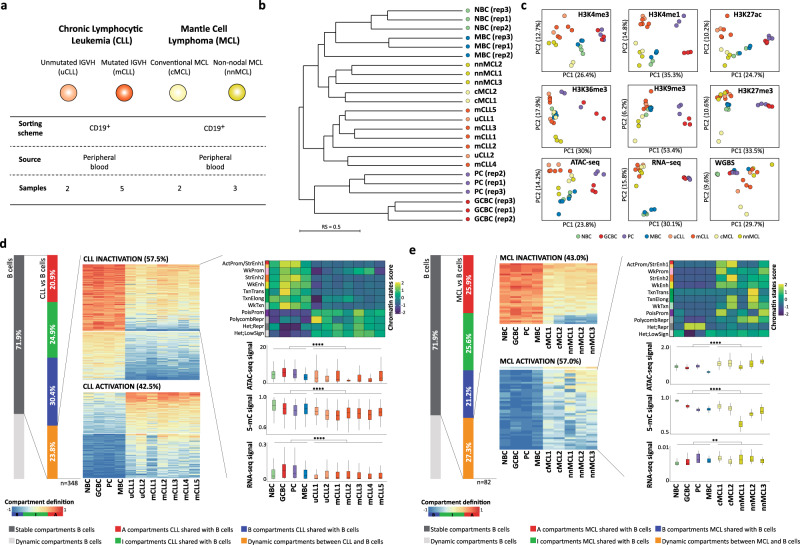
Characterization of the chromatin architecture of human B cell neoplasms. **a** Table of samples description together with the number of in situ Hi-C replicates analyzed by each CLL and MCL cases. **b** Dendrogram of the reproducibility scores for normalized Hi-C contact maps at 100 kb for B cell subpopulations replicates and samples from B cell neoplasia patients. IGHV unmutated (u)CLL; IGHV mutated (m)CLL; conventional (c)MCL and non-nodal (nn)MCL. **c** Unsupervised principal component analysis (PCA) for nine omics layers generated in the same patient samples as Hi-C: ChIP-seq of six histone marks (H3K4me3 *n* = 53,241 genomic regions, H3K4me1 *n* = 54,653 genomic regions, H3K27Ac *n* = 106,457 genomic regions, H3K36me3 *n* = 50,530 genomic regions, H3K9me3 *n* = 137,933 genomic regions, and H3K27me3 *n* = 117,560 genomic regions), chromatin accessibility measured by ATAC-seq (*n* = 140,187 genomic regions), DNA methylation measured by WGBS (*n* = 14,088,025 CpGs) and gene expression measured by RNA-seq (*n* = 57,376 transcripts). **d** Compartment changes upon CLL transformation. *Left*: First bar graph represents the percentage of stable and dynamic compartments during normal B cell differentiation. The second bar graph shows the percentage of stable and differential compartments in CLL as compared to normal B cells. A total of 23.8% of the compartments change in at least one CLL sample. *Middle*: Heatmaps showing eigenvector coefficients of the 348 compartments significantly losing (*n* = 200) or gaining (*n* = 148) activation in CLL samples as compared to normal B cells. *Right*: Multi-omics characterization of the 200 regions losing activity in CLL. Chromatin states, chromatin accessibility (ATAC-seq signal), DNA methylation (5-mC signal), and gene expression (RNA-seq signal) is shown for the CLL cases and normal B cells. The median signal of each layer was computed for NBC, GCBC, PC, and MBC while for the CLL the signal per each omics layer was considered. **e** Compartment changes upon MCL transformation. *Left*: First bar graph represents the percentage of stable and dynamic compartments in B cells. The second bar graph shows the percentage of conserved compartments between B cells and MCL. A total of 27.3% of the compartments change in at least one MCL sample. *Middle*: Heatmaps showing eigenvector coefficients of significant dynamic compartments between MCL and B cells (*n* = 82). Regions were split in two groups (MCL activation, *n* = 35 or inactivation, *n* = 47) according to the structural modulation of the MCL as compared to B cells. *Right*: Example of the MCL activation subset showing the chromatin states pattern, chromatin accessibility (ATAC-seq signal), DNA methylation (5-mC signal), and gene expression (RNA-seq signal). The median signal of each layer was computed for NBC, GCBC, PC, and MBC while for the MCL cases the signal per each omics layer was applied. Sample sizes were for uCLL: *n* = 2 biologically independent samples (all nine layers), for mCLL: *n* = 5 biologically independent samples (all nine layers), for cMCL: *n* = 2 biologically independent samples (all nine layers), for nnMCL: *n* = 3 biologically independent samples (all nine layers), for NBC, GCBC, PC, and MBC: *n* = 3 biologically independent samples (all nine layers with the exception of ATAC-seq for MBC in which *n* = 6 biologically independent samples were used). For the boxplots, centerline, box limits, and whiskers represent the median, 25th and 75th percentiles and 1.5× interquartile range, respectively. Comparisons were performed using the Wilcoxon rank-sum test (two-sided). **p value < 0.01, ****p value < 0.0001.

Next, we analyzed whether the observed 3D genome organization during normal B cell differentiation is further altered upon neoplastic transformation. To address this, we performed in situ Hi-C in fully characterized tumor cells from patients with CLL (*n* = 7) or MCL (*n* = 5). Within each neoplasm, we included cases of two subtypes, IGHV mutated (m, *n* = 5) and unmutated (u, *n* = 2) CLL as well as conventional (c, *n* = 2) and non-nodal leukemic (nn, *n* = 3) MCL (Fig. 4a and Supplementary Data 5). Initial unsupervised clustering of the RS from the entire Hi-C dataset indicated that CLL and MCL, similarly to the PCA from other omics layers generated from the same patient samples, clustered separately from each other and within a major cluster that included NBC and MBC (Fig. 4b, c, and Supplementary Fig. 3a). NBC and MBC have been described as potential cells of origin of these neoplasms^23^. Furthermore, pairwise eigenvector correlation analysis of the cancer samples suggested that the 3D genome configuration of the two clinico-biological subtypes of CLL was rather homogeneous (Supplementary Fig. 3b, c). This was not the case for the two MCL subtypes, which were more heterogeneous (Supplementary Fig. 3d, e).

The differential clustering of CLL and MCL samples hint into disease-specific changes of their 3D genome organization (Fig. 4b). To further detect those changes, we took the fraction of the genome with stable compartments during normal B cell differentiation and compared them to each lymphoid neoplasm. Qualitatively, we observed that roughly one-quarter of the genome changes compartments in at least one CLL (23.8%) and at least one MCL sample (27.3%) as compared to normal B cells (Fig. 4d, e left). Using a more stringent quantitative approach, we aimed at detecting changes associated with CLL or MCL as a whole, which revealed a total of 348 and 82 significant compartment changes (absolute difference in the eigenvalue > 0.4 and FDR < 0.05) in CLL and MCL, respectively. The larger number of regions changing compartments in CLL correlates with the results of the Hi-C based clustering (Fig. 4b), which indicates that MCL is more similar to NBC/MBC than CLL. Moreover, the observed compartment changes tended toward inactivation in CLL (57.5%) (Fig. 4d middle) and towards activation in MCL (57.0%) (Fig. 4e middle) compared to the normal B cells. These 3D genome organization changes were associated with the expected changes in chromatin function. Inactivation at the 3D genome level in CLL was linked to a shift to the poised promoter and polycomb-repressed chromatin states, and a significant loss of chromatin accessibility and gene expression (Fig. 4d right). Activation at the 3D genome level in MCL was accompanied by an enrichment of active chromatin states and a significant increase in chromatin accessibility and gene expression (Fig. 4e right). Overall, these results point to the presence of recurrent and specific changes in the 3D genome organization in CLL and MCL.

### *EBF1* downregulation in CLL is linked to extensive 3D genome reorganization

**Fig. 5:**
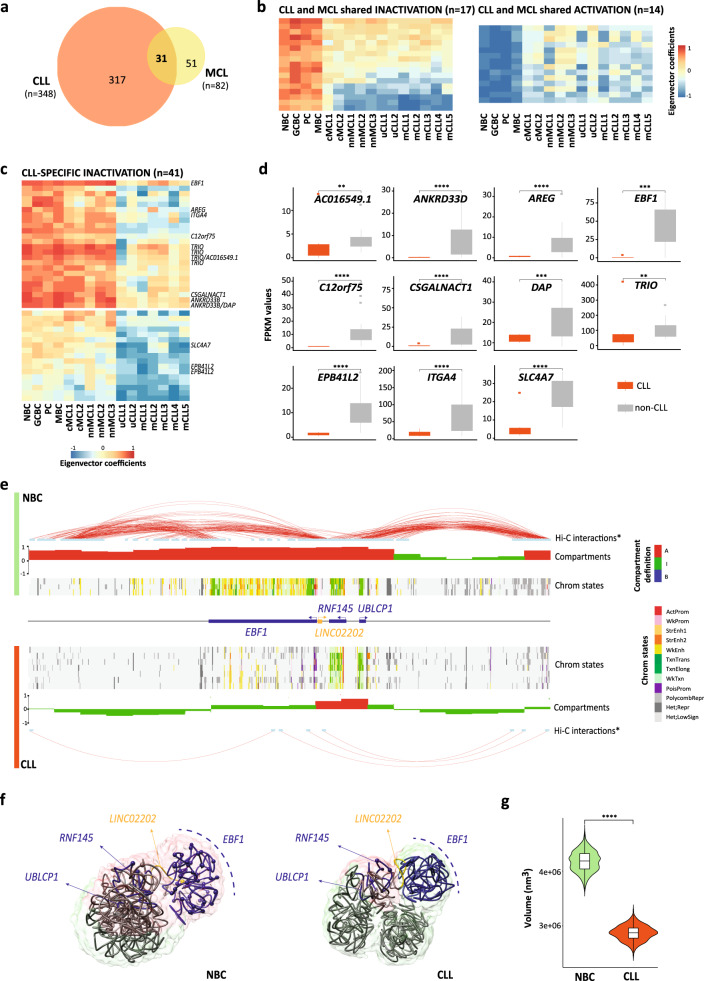
*EBF1* silencing in CLL is accompanied by structural changes affecting a 2 Mb region. **a** Venn diagram showing the significant number of dynamic compartments in CLL and MCL as compared to normal B cell differentiation and the regions shared between both B cell neoplasms (*n* = 31). **b** Heatmaps showing eigenvector coefficients of compartments significantly losing or gaining activation between B cell neoplasms (MCL and CLL together) and B cells. **c** Heatmap showing the eigenvector coefficients of the compartments losing activation specifically in CLL (*n* = 41). Significantly downregulated genes (FDR < 0.05) associated to each compartment are shown on the right of the heatmap. **d** FPKM values of all the CLL-specific significantly downregulated genes within compartments loosing activation. Sample sizes were for CLL: *n* = 7 biologically independent samples and for non-CLL: *n* = 17 biologically independent samples (*n* = 3 for each NBC, GCBC, PC, and MBC, *n* = 5 for MCL cases). **e** Map of the *EBF1* regulatory landscape. Significant Hi-C interactions (*p* value = 0.001) and compartment definitions from merged NBC and a representative CLL sample, followed by chromatin state tracks from each NBC (*n* = 3 biologically independent samples) and CLL (*n* = 7 biologically independent samples). The coordinates of the represented region are chr5:158,000,000–160,000,000, GRCh38. **f** Restraint-based model at 5 kb resolution of the 2 Mb region containing *EBF1* gene (total 400 particles, *EBF1* locus localized from 139 to 220 particle) in NBC and CLL. The surface represents the ensemble of 1000 models and is color-coded based on the compartment definition (A-type, B-type, and I-type in red, blue, and green, respectively). The top-scoring model is shown as trace, where protein-coding genes are colored in blue and long noncoding RNAs in yellow. Spheres represent enhancer regions. **g** Violin plot of the convex hull volume involving the 81 particles from the *EBF1* region. Sample sizes were for NBC: *n* = 3 biologically independent samples and for CLL: *n* = 7 biologically independent samples. For the boxplots, centerline, box limits, and whiskers represent the median, 25th, and 75th percentiles and 1.5× interquartile range, respectively. The comparison was performed using the Wilcoxon rank-sum test (two-sided). *adjusted *p* value < 0.05, **adjusted *p* value < 0.01, ***adjusted *p* value < 0.001, ****adjusted *p* value < 0.0001.

**Fig. 6:**
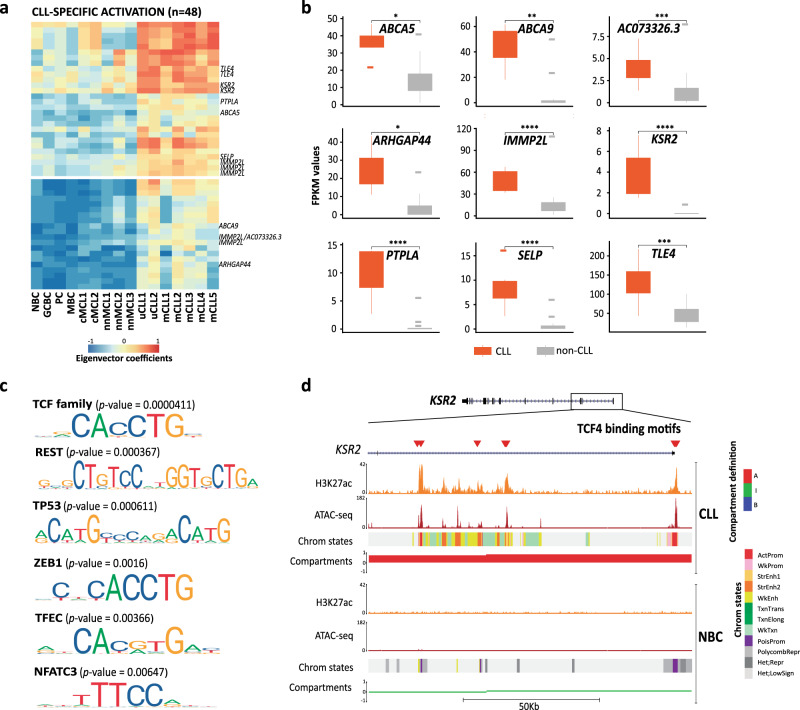
Transcription factors associated with CLL-specific activated compartments. **a** Heatmap showing the first eigenvector coefficients of the compartments gaining activation specifically in CLL (*n* = 48). Significantly upregulated genes (FDR < 0.05) associated with each compartment are shown on the right of the heatmap. **b** FPKM values of all the CLL-specific significantly upregulated genes within compartments gaining activation. **c** Enrichment analysis of transcription factor (TF) binding motifs. The most significant TF binding motifs enriched inactive and accessible loci within the CLL-specific regions gaining activity (*n* = 25 independent genomic loci) vs. the background (*n* = 28 independent genomic loci). *P* values were calculated using the Wilcoxon rank-sum test (one-sided). **d** Example of TCF4 binding motifs at the *KSR2* promoter region in CLL and NBC. The H3K27ac ChIP-seq signal, chromatin accessibility (ATAC-seq) and chromatin states of a representative NBC replicate and CLL sample are shown. The coordinates of the represented region are chr12:117,856,977–117,975,164, GRCh38. Sample sizes were for CLL: *n* = 7 biologically independent samples and for non-CLL: *n* = 17 biologically independent samples (*n* = 3 for each NBC, GCBC, PC, and MBC, *n* = 5 for MCL cases). For the boxplots, centerline, box limits, and whiskers represent the median, 25th, and 75th percentiles and 1.5× interquartile range, respectively. The comparison was performed using the Wilcoxon rank-sum test (two-sided). *adjusted *p* value < 0.05, **adjusted *p* value < 0.01, ***adjusted *p* value < 0.001, ****adjusted *p* value < 0.0001.

To further characterize the compartmentalization of neoplastic B cells, we classified the changing compartments as common (between CLL and MCL) or entity-specific (either in CLL or MCL). We detected 31 compartments commonly altered in both malignancies, revealing the existence of a core of regions that distinguish normal and neoplastic B cells (Fig. 5a, b). A targeted analysis of CLL and MCL revealed 89 CLL-specific (41 and 48 inactivated and activated, respectively) and only 3 MCL-specific compartment changes (Fig. 5c, Fig. 6a, and Supplementary Fig. 4a). The set of 41 compartments inactivated in CLL were significantly enriched (*p* value = 0.006) in downregulated genes (*n* = 11) as compared to normal B cells and MCL samples, being the early B cell factor 1 (*EBF1*) a remarkable example (Fig. 5c, d and Supplementary Data 6). *EBF1* downregulation has been described to be a diagnostic marker in CLL^50^, and its low expression may lead to reduced levels of numerous B cell signaling factors contributing to the anergic signature of CLL cells^51,52^ and low susceptibility to host immunorecognition^53,54^. To obtain insights into the mechanisms underlying *EBF1* silencing in CLL, we analyzed in detail a 2 Mb region hosting the gene, which also contains two nearby protein-coding genes, *RNF145* and *UBLCP1*, and a lncRNA, *LINC02202*. We observed that a large fraction of 3D interactions involving the *EBF1* region in normal B cells were lost in CLL resulting in a change from A-type to I-type compartment and a sharp inactivation of the gene, as shown by the analysis of chromatin states (Fig. 5e). Remarkably, in spite of the global reduction of 3D interactions, the two adjacent genes (*RNF145* and *UBLCP1*) were located in the only region (spanning 200 kb) that remained as A-type compartment in the entire 2 Mb region, maintaining thus an active state. To obtain further insights into the *EBF1* genome structure, we modeled its spatial organization in NBC and CLL by using the restraint-based modeling approach implemented in TADbit^55,56^ (Fig. 5f and Supplementary Fig. 4b, c). The *EBF1* domain in CLL resulted in larger structural variability as compared with the models in NBC due to the depletion of interactions in neoplastic cells (Supplementary Fig. 4b). The 3D models revealed that the *EBF1* gene is located in a topological domain, isolated from the rest of the region in NBC, hosting active enhancer elements (Fig. 5f). Remarkably, the active enhancer elements together with the interactions are lost in CLL (Fig. 5f), resulting in more collapsed conformations (Fig. 5g). Overall, these analyses suggest that *EBF1* silencing in CLL is linked to a compartment shift of a large genomic region leading to the abrogation of interactions and regulatory elements.

Our analysis also detected 48 regions that changed toward a more active compartment exclusively in CLL (Fig. 6a). As expected, these regions were significantly enriched in upregulated genes (*p* value = 0.0038) and harbored nine genes with increased expression (Fig. 6b and Supplementary Data 7). As previously shown for regions gaining activity in GCBC (Fig. 2e), we evaluated whether particular TFs were related to the CLL-specific increase in 3D interactions. Indeed, we found an enrichment in TF binding motifs of the TCF (*p* value = 0.00004) and NFAT (*p* value = 0.00647) families, which have been described to be relevant for CLL pathogenesis^29,57,58^ (Fig. 6c and Supplementary Data 8). One of the nine upregulated genes in CLL-specific active compartments was *KSR2*, a gene whose upregulation has a strong diagnostic value in CLL^50^. Importantly, this gene contained several motifs for the TCF4 TF (Fig. 6d), which itself is overexpressed in CLL as compared to normal B cells^29^, suggesting in this particular example that TCF4 overexpression may lead to aberrant binding to *KSR2* regulatory elements and a local remodeling of its 3D interactions.

### Increased 3D interactions across a 6.1 Mb region including the *SOX11* oncogene in aggressive MCL

**Fig. 7:**
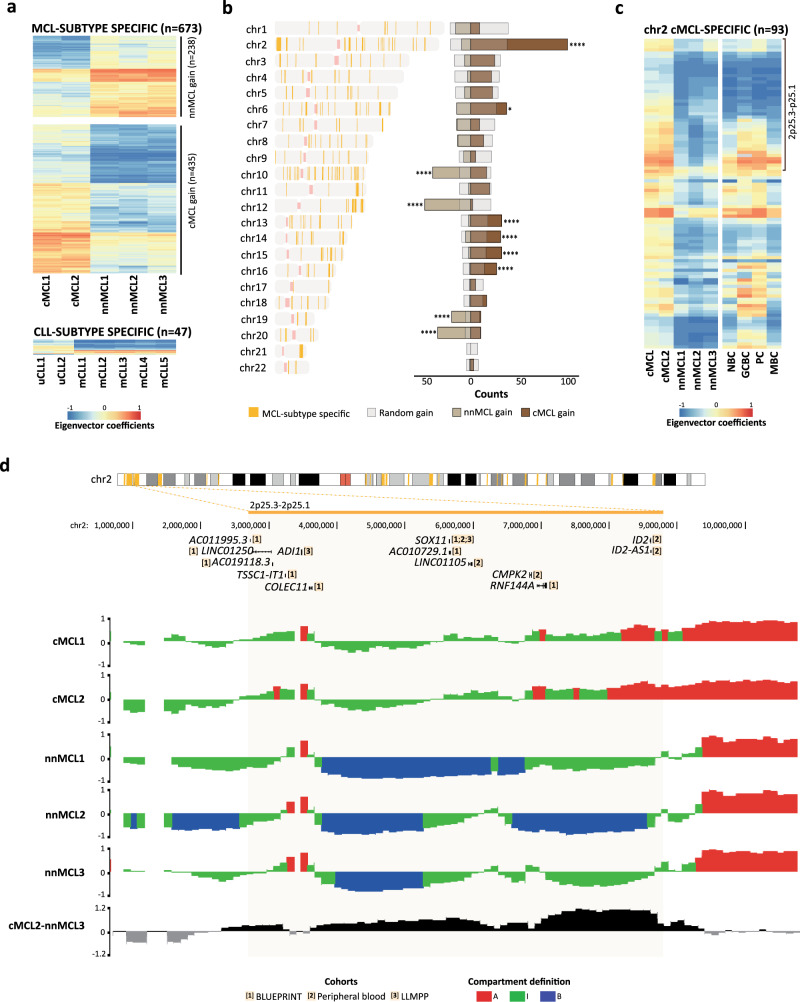
Long-range chromatin remodeling of a 6.1 Mb involving *SOX11* in cMCL. **a** Heatmaps showing eigenvector coefficients of compartments significantly changing in cMCL vs. nnMCL (*n* = 673) and in uCLL vs. mCLL (*n* = 47). **b** *Left*: Genome-wide distribution of compartments (its position is determined with orange vertical lines) changing in MCL subtypes. *Right*: Relative abundance of the compartments significantly gaining activity in cMCL or nnMCL as compared with a random probability evaluated using a Monte-Carlo test (one-tailed). A gain in compartment activation was defined as an increase of the eigenvector coefficient of at least 0.4. *p value < 0.05, ****p value < 0.0001. **c** Heatmap showing eigenvector coefficients of compartments specifically gaining activation in cMCL (*n* = 93) in chromosome 2. On top of the heatmap, a 6.1 Mb genomic block gaining activation in 2p25 is highlighted. **d** *Top*: Differentially expressed genes between cMCL and nnMCL in each of the three cohorts of transcriptional data of MCL patients. *Bottom*: Compartment definition tracks on all the MCL cases. Eigenvalue subtraction between a representative cMCL and an nnMCL cases highlighting the 6.1 Mb region gaining activity in the former.

In addition to entity-specific 3D genome changes, our initial analyses also suggested that different clinico-biological subtypes may have a different 3D genome organization, especially in MCL (Fig. 4b). To identify subtype differences within each B cell neoplasia, we selected regions with homogeneous compartments within each disease subtype and classified them as distinct if the difference between the Hi-C matrices cross-correlation eigenvalues was greater than 0.4. Applying this criterion, we defined 47 compartment changes between uCLL and mCLL, and 673 compartment changes between nnMCL and cMCL (Fig. 7a). This finding confirmed the previous analyses (Supplementary Fig. 3b–e) and indicated that the two MCL subtypes have a markedly different 3D genome organization. Two-thirds of the compartments changing in the MCL subtypes (*n* = 435, 64.6%) gained activity in the clinically aggressive cMCL, and one-third gained activity in nnMCL. We then characterized the chromosomal distribution of these compartment shifts, which, surprisingly, was significantly biased toward specific chromosomes (Fig. 7b). In particular, those regions gaining 3D interactions in aggressive cMCL were highly enriched in chromosome 2, being 22.3% (*n* = 97) of all 100 kb compartments located in that chromosome (Fig. 7b). We next analyzed chromosome 2 of cMCL in detail and we observed a de novo gain of A-type and I-type compartments accumulated at band 2p25 as compared to both normal B cells and nnMCL (Fig. 7c). The entire region of about 6.1 Mb had a dramatic increase of interactions and active chromatin states in cMCL as compared to nnMCL (Fig. 7d and Supplementary Fig. 5a). This region contains *SOX11*, whose overexpression in cMCL represents the main molecular marker to differentiate these two MCL subtypes^59^, and has been shown to play multiple oncogenic functions in cMCL pathogenesis^60–62^. However, as *SOX11* is embedded into a large block of 6.1 Mb gaining activation in cMCL, we wondered whether additional genes could also become upregulated as a consequence of the large-scale spatial organization of chromosomal band 2p25. Indeed, mining the expression data from the 5 MCL cases studied herein as well as two additional published cohorts^50,63^, we observed that 13 (43%) of the 30 expressed genes within the 6.1 Mb region were overexpressed in cMCL as compared to nnMCL in at least one cohort (Fig. 7d and Supplementary Fig. 5b–d), which may also contribute to cMCL pathogenesis and clinical aggressiveness.

## Discussion

We present a comprehensive analysis of the dynamic genome architecture reorganization during normal human B cell differentiation and upon neoplastic transformation into CLL and MCL. The integration of 3D genome data with nine additional omics layers including DNA methylation, chromatin accessibility, six histone modifications, and gene expression, has allowed us to obtain insights into 3D genome functional compartmentalization, cellular transitions across B cell differentiation, and 3D genome aberrations in neoplastic B cells. We initially explored the distribution of Hi-C eigenvector coefficients and identified that categorization into three components seemed to be more appropriate than the well-established dichotomous separation of the genome into A-type and B-type compartments^5^. Between the active (A) and repressed (B) compartments, we observed the presence of an intermediate (I) component which contained more inter-compartment interactions than fully active or inactive chromatin and is enriched in H3K27me3 located within poised promoters and polycomb-repressive chromatin states. This distribution resembles the traditional chromatin structure organization into euchromatin, facultative heterochromatin, and constitutive heterochromatin. In this context, the I-type compartment may represent the facultative heterochromatin, a labile state of the high-order chromatin organization that can evolve either into active or inactive chromatin compartments^64^. The I-compartment in part overlaps with the B1 compartment enriched in H3K27me3 described by Rao et al.^12^ in their recent classification of the 3D genome into six subcompartments^5^ (Supplementary Fig. 2e, f). A confirmation of the I-compartment may be further supported by several lines of published evidence. For example, during T cell commitment, a correlation between intermediate compartment scores with intermediate levels of gene expression was observed^15^. Recently, using super-resolution imaging, it was found that some compartments could belong to active or inactive states depending on the observed cell^65^, which could resemble an intermediate compartment in a population-based analysis such as Hi-C. Finally, this evidence is also in line with the observation that the polycomb repressive complex forms discrete subnuclear chromatin domains^66–68^ that can be dynamically modulated during cell differentiation^69,70^.

The three compartments had extensive modulation during human B cell differentiation, a process whose 3D genome architecture has been previously studied in cell lines and primary mouse cells^8,14,71–74^ or during the human germinal center reaction^16^. We observed that 28.1% of the genome is dynamically altered in particular B cell maturation transitions, a magnitude that is in line with compartment transitions observed during the differentiation of human embryonic stem cells into four cell lineages^7^ or the reprogramming of mouse somatic cells into induced pluripotent stem cells^8,75^, but lower than an analysis of compartment transitions across 21 human cells and tissues, which reached 59.6% of the genome^13^. The compartment modulation linked to B cell maturation was mainly related to two phenomena, a large-scale activation from NBC to GCBC and a reversion of the 3D genome organization of MBC back to the one observed in less mature NBC. As the number of mid-range 3D interactions upon activation has been suggested to decrease^76^, our result on the GCBC structural activation supports a previous study in which the chromatin structure of GCBC undergoes global de-compaction^16^. In this context, TFs have been described to act as the architects instructing structural changes in the genome^77^ and a recent report has described that TFs are able to drive topological genome reorganizations even before detectable changes in gene expression^8^. A detailed analysis of regions that become exclusively active in GCBC as compared to any other B cell subpopulation under study revealed enrichment in TF binding motifs of MEF2 and POU families, which have been described to play a key role in the germinal center formation^44^. In line with this important role of TFs in activating chromatin in GCBC, we also identified that NFAT and TCF binding motifs are enriched in those compartments specifically activated in CLL, and these TFs have also been previously linked to de novo active regulatory elements in CLL and its pathobiology^29^. All these results are concordant with studies in which lineage-restricted TFs have been proposed to establish and maintain genome architecture of specific lineages^14,77–79^. The outcome of the germinal center reaction is PC and MBC, which are phenotypically and functionally distinct subpopulations. GCBC and PC show an overall high level of conservation of their 3D genome organization, but the differentiation into MBC is related to extensive changes. Remarkably, we observed roughly three-quarters of the changes in MBC compartments reverted back to the compartment profile observed in NBC. This reversibility of the higher-order chromatin structure is very much in line with the previously observed similarity of histone modifications, chromatin accessibility, and gene expression profiles in NBC and MBC. In sharp contrast to this congruent behavior of chromatin-based traits, DNA methylation is rather different between NBC and MBC, as this mark follows an accumulative pattern during cell differentiation^31,80^ and can be used to faithfully track the lineage trajectory of the cells^81^.

We describe that B cell neoplasms show tumor-specific changes in the 3D genome organization that can span over large DNA stretches and contains genes linked to their pathogenesis. Of particular interest was the observation of the structural activation of 6.1 Mb affecting the entire chromosome band 2p25.2 in aggressive cMCL, which contains the *SOX11* oncogene, a biomarker whose expression defines this MCL subtype^59^ and plays key functional roles in its pathogenesis^82^. Although the *SOX11* oncogene expression is related to the presence of active histone modifications in the promoter region^83^ and the establishment of novel 3D loops with a distant enhancer element^33^, our finding indicates that such looping is embedded into long-range alterations in the 3D genome structure. This change is not only linked to *SOX11* overexpression but seems to be related to the simultaneous overexpression of multiple genes within the target region. This phenomenon of long-range epigenetic changes has been observed at the DNA methylation level, as the hypermethylation over one chromosomal band of 4 Mb has been linked to silencing of several genes in colorectal cancer^84^. In addition, in prostate cancer, long-range chromatin activation or inactivation analyzed by histone modifications has been shown to target oncogenes, microRNAs, and cancer biomarker genes^85^. The presence of large-range epigenetic remodeling in cancer^84–93^ shall support a more generalized use of genome-wide chromosome conformation capture techniques as part of the global characterization of primary human tumors. Beyond the identification of concerted deregulation of multiple contiguous genes with a potential role in cancer biology, targeting long-range aberrations in the 3D genome structure may itself represent a therapeutic target.

In conclusion, we provide an integrative and functional resource describing the dynamic 3D genome topology during human B cell differentiation and neoplastic transformation. Our analysis points to a highly dynamic 3D genome organization in normal B cells, including extensive activation from NBC to GCBC and reversibility in MBC. In neoplastic cells from CLL and MCL, we identify the disease and subtype-specific change in the 3D genome organization, which include large chromatin blocks containing genes playing key roles in their pathogenesis and clinical behavior.

## Methods

### Isolation of B cell subpopulations for in situ Hi-C experiment

Four B cell subpopulations spanning mature normal B cell differentiation were sorted for in situ Hi-C as previously described^31^. Briefly, peripheral blood B cell subpopulations, i.e., NBC and MBC were obtained from buffy coats for healthy adult male donors from 56 to 61 years of age, obtained from Banc de Sang i Teixits (Catalunya, Spain). GCBC and PC were isolated from tonsils of male children undergoing tonsillectomy (from 2 to 12 years of age), obtained from the Clínica Universidad de Navarra (Pamplona, Spain). Samples were cross-linked before FACS sorting, to separate each of the B cell subpopulations, and afterward were snap-frozen and kept at −80 °C. Three replicates per B cell subpopulation were processed and each replicate was derived from individual donors with the exception of PC, for which two of the three replicates proceeded from the pool of four different donors. The use of the samples analyzed in the present study was approved by the ethics committee of the Hospital Clínic de Barcelona and Clínica Universidad de Navarra.

### Patient samples

The samples from CLL (*n* = 7)^29^ and MCL (*n* = 5) patients were obtained from cryopreserved mononuclear cells from the Hematopathology collection registered at the Biobank (Hospital Clínic-IDIBAPS; R121004-094). All samples were >85% tumor content. Clinical and biological characteristics of the patients are shown in Supplementary Data 5.

The enrolled patients or legally authorized representatives/parents minor participants (age below 18 years of age) gave informed consent for scientific study following the ICGC guidelines and the ICGC Ethics and Policy committee^94^. This study was approved by the clinical research ethics committee of the Hospital Clínic of Barcelona.

### In situ Hi-C

In situ Hi-C was performed based on the previously described protocol^12^. Two million cross-linked cells per sample were used as starting material. Chromatin was digested adding 100U DpnII (New England BioLabs) and incubated overnight. After the fill-in with bio-dCTP (Life-Technologies, 19518-018), nuclei were centrifuged 5 min, 3000 rpm at 4 °C, and ligation was performed for 4 h at 16 °C adding 2 µl of 2000 U/µl T4 DNA ligase on a total of 1.2 mL of ligation mix (120 µl of 10× T4 DNA ligase buffer; 100 µl of 10% Triton X-100; 12 µl of 10 mg/ml BSA; 966 µl of H_2_O). Following ligation, nuclei were pelleted and resuspended with 400 µl 1× NEBuffer2 (New England BioLabs). Then, 10 µl of RNAseA (10 mg/ml) was added to the nuclei and incubated for 15 min at 37 °C while shaking (300 rpm), and after that 20 µl of proteinase K (10 mg/mL) was added and incubated overnight at 65 °C while shaking (600 rpm). After reversion of the cross-link, DNA was purified by phenol/chloroform/isoamyl alcohol and DNA was precipitated by adding to the upper aqueous phase: 0.1× of 3 M sodium acetate pH 5.2, 2.5× of pure ethanol, and 50 µg/ml glycogen. Samples were mixed and incubated overnight at −80 °C. Next, samples were centrifuged 30 min at 13,000 rpm at 4 °C, and the pellet was washed with 1 mL of EtOH 70% followed by a 15 min centrifugation at 13,000 rpm at 4 °C. The supernatant was discarded and the pellet air-dried for 5 min and resuspended in 130 µl of 1× Tris buffer (10 mM TrisHCl, pH 8.0), which to be fully dissolved was incubated at 37 °C for 15 min. Purified DNA was sonicated using Covaris S220, and then the final volume was adjusted to 300 µl with 1× Tris buffer. Sonicated DNA was mixed with washed magnetic streptavidin T1 beads (total of 100 µl 10 mg/ml beads), split in two tubes (150 µl each), and incubated for 30 min at room temperature (RT) under rotation. Subsequently, beads were separated on the magnet, the supernatant discarded and the DNA was washed with 400 µl of BB 1×, twice. Sonicated DNA conjugated with beads was washed with 100 µl of 1× T4 DNA ligase buffer, pooling the two tubes per condition. After that, beads were reclaimed in the end-repair mix. Once incubated for 30 min at RT the beads were washed twice with 400 µl of BB 1×. Then, beads were washed with 100 µl of NEBuffer2 and reclaimed in A-tailing mix, incubated for 30 min at 37 °C and washed twice with 400 µl of BB 1×, followed by a wash in 100 µl of 1× T4 DNA ligase buffer. Afterward, the beads were resuspended in 50 µl of 1× Quick ligation buffer, 2.5 µl of Illumina adaptors, and 4000 U of T4 DNA ligase and incubated for 15 min at RT. Then, beads were washed twice with 400 µl BB 1× and resuspended in 30 µl of 1× Tris buffer. In the end, libraries were amplified by eight cycles of PCR using 8.3 µl of beads and pooling a total of 4 PCRs per sample. The PCR products were mixed by pipetting with an equal volume of AMPure XP beads and incubated at RT for 5 min. Beads were washed with 700 µl of EtOH 70%, without mixing, twice, and left the EtOH to evaporate at RT without over-drying the beads (approximately 4 min). Finally, the beads were resuspended with 30 µl 1× Tris buffer, incubated for 5 min, and the supernatant containing the purified library was transferred in a new tube and stored at −20 °C. DNA was quantified by Qubit dsDNA High Sensitivity Assay, the library profile was evaluated on the Bioanalyzer 2100 and the ligation was assessed. Libraries were sequenced on HiSeq 2500. Supplementary Data 1 summarizes the number of reads sequenced and quality metrics for each B cell subpopulation replicate and B cell neoplasm.

### Hi-C data preprocessing, normalization, and interaction calling

The sequencing reads of Hi-C experiments were processed with TADbit version 0.4.62^56^. Briefly, sequencing reads were aligned to the reference genome (GRCh38) applying a fragment-based strategy; dependent on GEM mapper^95^. The mapped reads were filtered to remove those resulting from unspecified ligations, errors, or experimental artifacts. Specifically, we applied seven different filters using the default parameters in TADbit: self-circles, dangling ends, errors, extra dangling-ends, over-represented, duplicated, and random breaks^56^. Hi-C data were normalized using the OneD R package version 0.0.0.9100^96^ at 100 kb of resolution to remove known experimental biases, a method that controls for the presence of abnormal karyotypes in cancer samples^96^. The significant Hi-C interactions were called with the *analyzeHiC* function of the HOMER software suite version 4.9.1^78^, binned at 10 kb of resolution and with the default *p* value threshold of 0.001.

### Reproducibility of Hi-C replicas

The agreement between Hi-C replicates was assessed using the RS^37^. The RS is a measure of matrix similarity ranging between 0 (totally different matrices) and 1 (identical matrices). A genome-wide RS was defined for each experiment as the average RS between pairs of corresponding normalized chromosome matrix (Supplementary Fig. 1a and Supplementary Fig. 3a). Then, the matrix representing all the genome-wide RSs was analyzed using a hierarchical clustering algorithm with Ward’s agglomeration method using *hclust* function from stats R package version 3.5.1.

### ChIP-seq and ATAC-seq data generation and processing

ChIP-seq of the six different histone marks and ATAC-seq data were generated as described in (http://www.blueprint-epigenome.eu/index.cfm?p=7BF8A4B6-F4FE-861A-2AD57A08D63D0B58)^29^. Briefly, fastq files of ChIP-seq data were aligned to the GRCh38 reference genome using bwa version 0.7.7^97^, Picard version 2.8.1 (http://broadinstitute.github.io/picard/) and SAMtools version 1.3.1^98^, and wiggle plots were generated (using PhantomPeakQualTools R package version 1.1.0) as described (http://dcc.blueprint-epigenome.eu/#/md/methods). Peaks of the histone marks were called as described in http://dcc.blueprint-epigenome.eu/#/md/methods using MACS2 version 2.0.10.20131216^99^ with input control. ATAC-seq fastq files were aligned to genome build GRCh38 using bwa version 0.7.7 (parameters: -q 5 –P -a 480)^97^ and SAMtools version 1.3.1 (default settings)^98^. BAM files were sorted and duplicates were marked using Picard tools version 2.8.1 with default settings (http://broadinstitute.github.io/picard/). Finally, low quality and duplicate reads were removed using SAMtools version 1.3.1 (parameters: -b -F 4 -q 5,-b, -F 1024)^98^. ATAC-seq peaks were determined using MACS2 version 2.1.1.20160309(parameters: -g hs q 0.05 -f BAM –nomodel - shift −96 extsize 200 - keep -dup all) without input^99^. For downstream analyses peaks with FDR threshold of 1 × 10^−7^ (H3K4me3, H3K4me1, H3K27ac) or 1 × 10^−2^ (H3K36me3, H3K9me3, H3K27me3, and ATAC-seq) were included.

For each mark a set of consensus peaks (chr1-22) present in the normal B cells (*n* = 12 biologically independent samples for histone marks and *n* = 15 biologically independent samples for ATAC-seq) was generated by merging the locations of the separate peaks per individual sample. Also, the second set of consensus peaks was generated taking into account normal B cells, CLL (*n* = 7 biologically independent samples), and MCL (*n* = 5 biologically independent samples). For the histone marks, the number of reads per sample per consensus peak was calculated using the *genomecov* function of BEDtools suite version 2.25.0^100^. For ATAC-seq, the number of insertions of the TN5 transposase per sample per consensus peaks was calculated determining the estimated insertion sites (shifting the start of the first mate 4 bp downstream), followed by the *genomecov* function of BEDtools suite version 2.25.0^100^. The number of consensus peaks for normal B cell samples were 46,184 (H3K4me3), 44,201 (H3K4me1), 72,222 (H3K27ac), 25,945 (H3K36me3), 40,704 (H3K9me3), 20,994 (H3K27me3), 99,327 (ATAC-seq), while the number of consensus peaks for normal B cells, CLL and MCL samples were 53,241 (H3K4me3), 54,653 (H3K4me1), 106,457 (H3K27ac), 50,530 (H3K36me3), 137,933 (H3K9me3), 117,560 (H3K27me3), 140,187 (ATAC-seq). Using DESeq2 R package version 1.28.0^101^ counts for all consensus peaks were transformed by means of the variance stabilizing transformation (VST) with blind dispersion estimation. PCAs were generated with the *prcomp* function from the stats R package version 3.5.1 using the VST values.

### RNA-seq data generation and processing

Single-stranded RNA-seq data were generated as previously described^102^. Briefly, RNA was extracted using TRIZOL (Life Technologies) and libraries were prepared using TruSeq Stranded Total RNA kit with Ribo-Zero Gold (Illumina). Adapter-ligated libraries were amplified and sequenced using 100 bp single-end reads. RNA-seq data of the 24 samples, some (*n* = 19) mined from a previous study^29^, were aligned to the reference human genome build GRCh38 (Supplementary Data 5). Signal files were produced and gene quantifications (gencode 22, 60,483 genes) were calculated as described (http://dcc.blueprint-epigenome.eu/#/md/methods) using the GRAPE2 pipeline with STAR version 2.4.0j and RSEM version 1.2.21 software (adapted from the ENCODE Long RNA-Seq pipeline). The expected counts and fragments per kilobase million (FPKM) estimates were used for downstream analysis. The PCA of the RNA-seq data was generated with the *prcomp* function from the stats R package version 3.5.1 in the 12 analyzed normal B cell samples or 24 analyzed normal and neoplastic B cell samples.

### WGBS data generation and processing

WGBS was generated as previously described^31^. Mapping and determination of methylation estimates were performed as described (http://dcc.blueprint-epigenome.eu/#/md/methods) using GEM version 3.0. Per sample, only methylation estimates of CpGs with ten or more reads were used for downstream analysis. The PCA of the DNA methylation data was generated with the *prcomp* function from the stats R package version 3.5.1 using methylation estimates of 15,089,887 CpGs (chr1-22) with available methylation estimates in all 12 analyzed normal B cell samples or 14,088,025 CpGs (chr1–22) in all 24 analyzed normal and neoplastic B cell samples.

### Definition of sub-nuclear genome compartmentalization

The segmentation of the genome into compartments was determined as previously described^5^. In short, normalized chromosome-wide interaction matrices at 100 kb resolution were transformed into Pearson correlation matrices. These correlation matrices were then used to perform PCA for which the first eigenvector (EV) normally delineates genome segregation. All EVs were visually inspected to ensure that the EV selected corresponded to genomic compartments^5^. For a limited number of chromosomal regions (i.e. chromosome 19 for MBC-rep2, uCLL2, mCLL2, and mCLL4 as well as chromosomes 13 and 18 for uCLL2), TADbit could not unequivocally assign compartments due to the sparseness of the Hi-C datasets. Since the sign of the EV is arbitrary, a rotation factor based on the histone mark H3K4me1 signal and ATAC-seq signal were applied to correctly call the identity of the compartments. A Pearson correlation coefficient was computed between the EVs for each pair of merged B cell subpopulation (Supplementary Fig. 1c). Each merged sample was also correlated with its replica (Supplementary Fig. 1c). The multi-modal distribution of the EV coefficients from the B cells dataset was modeled as a Gaussian mixture with three components (*k* = 3). To estimate the mixture distribution parameters, an Expectation-Maximization algorithm using the *normalmixEM* function from the mixtools R package version 1.2.0 was applied^103^.

A Bayesian Information Criterion (BIC) was computed for the specified mixture models of clusters (from 1 to 10) using *mclustBIC* function from mclust R package version 5.4.6^104^ (Supplementary Fig. 1d). Three underlying structures were defined; alternative compartmentalization into A-type (with the most positive EV values), B-type (with the most negative EV values), and I-type (an intermediate-valued region with a distinct distribution) compartments. Two intersection values (IV1, IV2) were defined at the intersection points between two components. The mean IV1 and IV2 values across all the B cell replicas (*n* = 12) were then used as standard thresholds to categorize the data into the three different components (that is, A-type compartment was defined for EV values between +1.00 and +0.43, I-type compartment was defined for EV values between +0.43 and −0.63, and B-type compartment was defined for EV values between −0.63 and −1.00) (Supplementary Fig. 1e).

### Characterizing compartment types in B cells by integrating nine omics layers

Given a set of peaks as previously defined by Beekman et al.^29^ from nine different omics layers including six histone marks (H3K4me3, H3K4me1, H3K27ac, H3K36me3, H3K9me3, and H3K27me3), gene accessibility (ATAC-seq), gene expression (RNA-seq), and DNA methylation (WGBS), a *bedmap* function from BEDOPS software version 2.4.28^105^ was applied to get the mean scoring peak over the 100 kb intervals genome-wide. Next, Pearson correlation coefficients were computed between the EV coefficients and the mean scoring value of each epigenetic mark at 100 kb intervals (Supplementary Fig. 1b). Finally, the mean scoring values were normalized by the total sum of the values for each mark and grouped by the three defined genomic compartments (A, I, B-type; Fig. 1g). A Wilcoxon rank-sum test was used to compute the significance between all the possible pairwise comparisons of the signal distribution.

### Compartment interaction score (C-score)

The compartment score is defined as the ratio of contacts between regions within the same compartment (intra-compartment contacts) over the total chromosomal contacts per compartment (intra-compartment + inter-compartment). To compute the compartment score, all the compartments that shared the same genomic segmentation were merged.

### Chromatin states enrichment by genomic compartments

The genome was segmented into 12 different chromatin states at 200 bp interval as previously described^29^. The active promoter and strong enhancer 1 were merged as a unique state, giving a total of 11 chromatin states. The genome compartmentalization was next split into 4 groups; 3 conserved groups, in which the B cells samples shared A-type compartment (*n* = 6,409), B-type compartment (*n* = 6,267), or I-type compartment (*n* = 5,467) and a dynamic group (*n* = 7,099) of non-conserved compartmentalization among B cells subpopulation. Each group was correlated with the defined 11 chromatin states using *foverlaps* function from data.table R package version 1.13.0. The frequency of each chromatin state (corrected by the total frequency in the genome) was computed per each genomic compartment. The chromatin state score is thus the median frequency of the three replicas scaled by the columns and the rows using *scale* function from base R package version 3.5.1.

### Description of chromatin states in the intermediate (I)-type compartment

A 200 bp-windows containing poised promoter (*n* = 547) or polycomb-repressed (*n* = 11,665) chromatin states were extracted from the NBC intermediate compartments (*n* = 1,885). From those regions, two main subgroups were distinguished according to the chromatin state shown in the next stage of differentiation (GCBC): (1) those regions that maintained their chromatin state (poised promoter or polycomb-repressed), and (2) those regions that changed their chromatin state; which were further classified into three categories: (i) I-related chromatin states (poised promoter or polycomb-repressed), (ii) B-related chromatin states (repressive heterochromatin and low signal heterochromatin), (iii) A-related chromatin states (active promoter/strong enhancer 1, weak promoter, strong enhancer 2, transcription transition, transcription elongation, and weak transcription). Finally, the fold-change of related chromatin states between GCBC and NBC was computed.

### Analysis of chromatin state dynamics upon B cell differentiation

B cell differentiation axis was divided into two main branches: (i) NBC-GCBC-PC and (ii) NBC-GCBC-MBC. Both branches presented a common step from NBC to GCBC and then a divergence step in PC or MBC. The 5,445 common compartments from both branches were considered for the analysis. The general modulation of chromatin structure was drawn using the *alluvial* function from alluvial R package version 0.1.2.

### TF analyses

From GCBC-specific 937 active compartments (B to A-type, *n* = 18; B to I-type, *n* = 512 and I to A-type, *n* = 407) were narrowed down to 171 peaks due to the following filtering steps: (i) only the 200 bp-windows contain an active promoter, strong enhancer 1, and strong enhancer 2 chromatin states were retained (*n* = 1,907 regions). (ii) Regions, where H3K27ac peaks were differentially enriched in GCBC, replicates compared to the rest of normal B cell subpopulations (FDR < 0.05) computed using DESeq2 R package version 1.28.0^101^ were retained. (iii) Regions with a presence of ATAC-seq peaks in at least two GCBC replicates were retained (*n* = 171 peaks). The background considered as the rest of the ATAC-seq peaks (*n* = 268) presented at the 1,907 regions in at least two GCBC replicates.

From CLL-specific 48 active compartments (in normal B cells defined as I-type: *n* = 28 and B-type: *n* = 20), were narrowed down to 25 peaks due to the following filtering steps: (i) regions where H3K27ac peaks were differentially enriched (FDR < 0.05) comparing CLL from all normal B cells and MCL using DESeq2 R package version 1.28.0^101^, (ii) regions where ATAC-seq peaks were presented in at least five CLL (*n* = 25). The background considered was all the resting ATAC-seq peaks (*n* = 28) on the 48 compartments presented in at least five CLL.

On both analyses, FASTA sequences of targeted regions (GCBC-specific regions and CLL-specific regions) were extracted using *getfasta* function from BEDtools suite version 2.25.0^100^ using GRCh38 as reference assembly. An analysis of motif enrichment was done by the AME-MEME suite version 5.0.3^106^ using non-redundant TF-binding profiles of *Homo sapiens* Jaspar 2018 database^107^ as a reference motif database. The database contained a set of 537 DNA motifs. Maximum odd scores were used as a scoring method and one-tailed Wilcoxon rank-sum test as motif enrichment test. Only TF genes that were expressed (FPKM median values > 1) were included.

### TCF4-binding motif example from the *KSR2* gene

FASTA sequences of 25 ATAC-seq peaks detected in CLL-specific active compartments were extracted using GRCh38 as reference assembly. A search of individual motif occurrences analysis was done using AME-FIMO suite version 4.12^108^ library(BSgenome.Hsapiens.UCSC.hg38,masked) with a custom random model (letter frequencies: A, 0.262: C, 0.238: G, 0.238 and T, 0.262). A *p* value < 0.0001 was established as a threshold to determine 23 significant motif occurrences where TCF4 binding motif (MA0830.1) was one of the top candidates.

### Log-ratio of normalized interactions in the *AICDA* regulatory landscape

Normalized Hi-C maps were analyzed at 50 kb of resolution at the specific genomic region, chr12:8,550,000–9,050,000 (GRCh38), from the four B cell subpopulations. A logarithmic ratio of the contact maps was computed between NBC and GCBC and GCBC with PC and MBC. The resulting array was convolved with a one-dimensional Gaussian filter of standard deviation (sigma) of 1.0 using and interpolated with a nearest-neighbor approach using scipyndimage Python package version 1.2.1.

### Statistical testing for detecting significantly changed compartment regions

Briefly, 100 kb regions that had at least one missing value among the compared samples were removed from the analysis. Then, two different groups were defined, case and control, according to the case–control pair analyzed. A *t* test was computed to compare each case–control pair, and the resulting *p* values were adjusted using the false discovery rate (FDR)^109^. The regions with significantly different means and fold changes were selected based on two specific thresholds: a *p*-adjustment value less than 0.05 and a fold change greater than 0.4. The results were then generated for a total of four different case–control pairs.

(I) Control: all regions conserved across all B cell samples without missing values in CLL (A-type, *n* = 3,967, I-type, *n* = 4,301 and B-type, *n* = 5,226), case: all CLL regions non-conserved in B cell samples (*n* = 3,217). The analysis resulted in 348 B cell_CLL significantly changed regions.

(II) Control: all regions conserved across all B cell samples without missing values in MCL (A-type *n* = 6,167, I-type *n* = 5,299, B-type *n* = 5,812), case: all MCL regions non-conserved in B cell samples (*n* = 4,716). The analysis resulted in 82 B cell_MCL significantly changed regions.

(III) Control: B cell-CLL significantly changed regions (*n* = 348) — MCL-CLL overlapping (*n* = 31) = B cell-CLL specific regions (n = 317), case: MCL regions (A-type *n* = 97, I-type *n* = 154, B-type *n* = 61; total *n* = 312). The analysis resulted in 89 B cell_CLL-specific regions.

(IV) Control: B cell-MCL significantly changed regions (*n* = 82) — MCL-CLL overlapping (*n* = 31) = B cell-MCL specific regions (*n* = 51), case: CLL regions (*n* = 41). The analysis resulted in three B cell_MCL-specific regions.

### Integrative 3D modeling of EBF1 and structural analysis

Hi-C interactions matrices from the merging of three replicas of NBC and the seven cases of CLL were used to model chr5:158,000,000:160,000,000 (GRCh38) at 5 kb of resolution. For NBC and CLL merged Hi-C interaction maps, an MMP score was calculated to assess the modeling potential of the region, resulting in 0.79 for NBC and 0.84 for CLL indicative of good quality Hi-C contact maps for accurate 3D reconstruction^110^. Next, this region was modeled using a restraint-based modeling approach as implemented in TADbit version 0.4.62^56^, where the experimental frequencies of interaction are transformed into a set of spatial restraints^55^. Briefly, each 5 kb bin of the interaction Hi-C map was represented as a spherical particle in the model, which resulted in 400 particles each of radius equal to 25 nm. All the particles in the models were restrained in the space based on the frequency of the Hi-C contacts, the chain connectivity, and the excluded volume. The TADbit optimal parameters (maxdist = −1.0; lowfreq = 1.0; upfreq = 200; and dcutoff = 150) resulted in the best Spearman correlations of 0.61 (NBC) and 0.63 (CLL) between the Hi-C interaction map and the model’s contact map. Next, a total of 5000 models per cell type were generated, and the top 1000 models that best satisfied the imposed restraints were retained for the analysis. To assess the structural similarities among the 3D models, the distance root-mean-square deviations (dRMSD) value was computed for all the possible pairs of top models (1000 in NBC and 1000 in CLL) and a hierarchical clustering algorithm was applied on the resulting dRMSD matrix using *ward.D* method from stats R package version 3.5.1 (Supplementary Fig. 4c). The convex hull volume spanned by the 81 particles of the *EBF1* gene (chr5:158,695,000–159,000,000, GRCh38) was computed in each model using the *convexhull* function from the scipy.spatial Python package version 1.2.1 (Fig. 5g).

### Differential Gene expression analyses

Differentially expressed genes were defined using the DESeq2 R package version 1.28.0^101^ in all the genes. Then, the genes present in the compartments of interest were selected and the Benjamini y Hochberg test (FDR < 0.05) was applied. In detail, expected counts were used on the following considered comparisons: (i) for GCBC-specific activate compartments, GCBC samples (*n* = 3) vs. the rest of normal B cells samples (NBC, PC, MBC; *n* = 9); (ii) for CLL-specific active compartments, CLL samples (*n* = 7) vs. the rest of the samples (normal B cells and MCL, *n* = 17); (iii) for CLL-specific inactive compartments, all normal B cells and MCL samples (total *n* = 17) vs. CLL samples (*n* = 7), and (iv) for cMCL, cMCL (*n* = 2) vs. nnMCL (*n* = 3) samples were studied. Then, the expression of the genes differentially expressed on each comparison of interest was assessed. Only genes that were expressed (FPKM median values > 1) were included.

The *findOverlaps* function from GenomicRanges R package version 1.34.0^111^ was used to annotate genes that overlapped with these defined regions. The one-tailed Monte–Carlo method was applied to evaluate the significant number of differentially expressed genes in CLL-specific compartments (this process was randomly repeated 10,000 times).

### Defining de novo (in)active regions in sub-type specific neoplastic group

MCL and CLL patient samples were grouped according to their biological and clinical characteristics. This classification resulted in two conventional (c) and three leukemic non-nodal (nn) MCL cases and two IGHV-unmutated (u) and five IGHV-mutated (m) CLL cases.

First, the non-assigned neoplasia compartments were removed from the analysis. A sample homogenization was applied to reduce the intra-subtype variance; the samples that presented a difference of EV smaller than 0.4 were retained (91.3% in MCL, 87.1% CLL). Next, to study the inter-subtype variance, the mean of the EV from each subtype of B cell malignancy was computed. Significant regions were determined if the difference between the two subtypes (cMCL vs. nnMCL and uCLL vs. mCLL) was equal or higher than 0.4, which resulted in 673 regions in MCL and 47 in CLL. MCL-subtype specific regions were split into two groups according to the value of its EV coefficient (*n* = 435 region called cMCL gain, *n* = 238 regions called nnMCL gain). The distribution and the frequency of the significantly changed regions were studied per chromosome and compared with the probability of finding them by chance in each chromosome. N-subsamples of 100 kb size was selected from the GRCh38 genome and their frequency was calculated per chromosome (this process was randomly repeated 10,000 times). The one-tailed Monte–Carlo method was applied to compute *p* values. The *findOverlaps* function from GenomicRanges R package version 1.34.0^111^ was next used to annotate protein-coding genes that overlapped with these defined regions. Differentially expressed genes among cMCL and nnMCL at chr2:2,700,000–8,800,000 (GRCh38) was computed using DESeq2 R package version 1.28.0^101^ (using a FDR < 0.05). The expression analysis was validated in two independent published cohorts, i.e., a series with 30 conventional and 24 leukemic non-nodal MCL (GEO GSE79196) from peripheral blood^50^ and a second series from the lymphoma/leukemia molecular profiling project (LLMPP) (GEO GSE93291)^63^. The microarrays were normalized using the frma R package version 1.38.0^112^ and limma R package version 3.42.2^113^ was used to identify differentially expressed genes with adjusted *p* value < 0.05. Standardized expression matrices were used to do the heatmaps using pheatmap R package version 1.0.12. Gene differentially expressed on the identified cohort: [1] RNAseq from BLUEPRINT data, [2] peripheral blood, and [3] LLMPP. The magnitude of the compartmentalization change was calculated by subtracting the EV of cMCL1 and nnMCL2. The karyotype and chromosome 2 were designed using the karyoploteR package version 1.14.1^114^.

### Reporting summary

Further information on experimental design is available in the Nature Research Reporting Summary linked to this paper.

## Supplementary information

Supplementary Information

Peer Review File

Description of Additional Supplementary Files

Supplementary Data 1

Supplementary Data 2

Supplementary Data 3

Supplementary Data 4

Supplementary Data 5

Supplementary Data 6

Supplementary Data 7

Supplementary Data 8

Reporting Summary

## Data Availability

In situ Hi-C data generated within this study have been deposited at the European Genome-Phenome Archive (EGA, http://www.ebi.ac.uk/ega/), which is hosted at the European Bioinformatics Institute (EBI), accession number EGAS00001004763. The remaining epigenomic data from normal B cells, CLL and MCL generated within the Blueprint Consortium can be found under accession numbers EGAS00001000326 (ChIP-seq), EGAS00001001596 (ATAC-seq), EGAS00001000418 (WGBS) and EGAS00001000327 (RNA-seq). We have created a website accompanying the manuscript [http://resources.idibaps.org/paper/dynamics-of-genome-architecture-and-chromatin-function-during-human-b-cell-differentiation-and-neoplastic-transformation], which contains links to ucsc sessions displaying the multi-omics data, to the *EBF1* models using TADkit, and to the Hi-C matrices at 20 kb resolution. All other relevant data supporting the key findings of this study are available within the article and its Supplementary Information files or from the corresponding author upon reasonable request. A reporting summary for this article is available as a Supplementary Information file.
