# Supplementary material for: Dynamics of genome architecture and chromatin function during human B cell differentiation and neoplastic transformation
Source: Nat Commun. 2021 Jan 28;12:651. doi: 10.1038/s41467-020-20849-y (PMC7844026; doi:10.1038/s41467-020-20849-y)
Supplement: Supplementary file 1 — Supplementary Information [file 41467_2020_20849_MOESM1_ESM.pdf]

Supplementary Figures

Supplementary Fig. 1

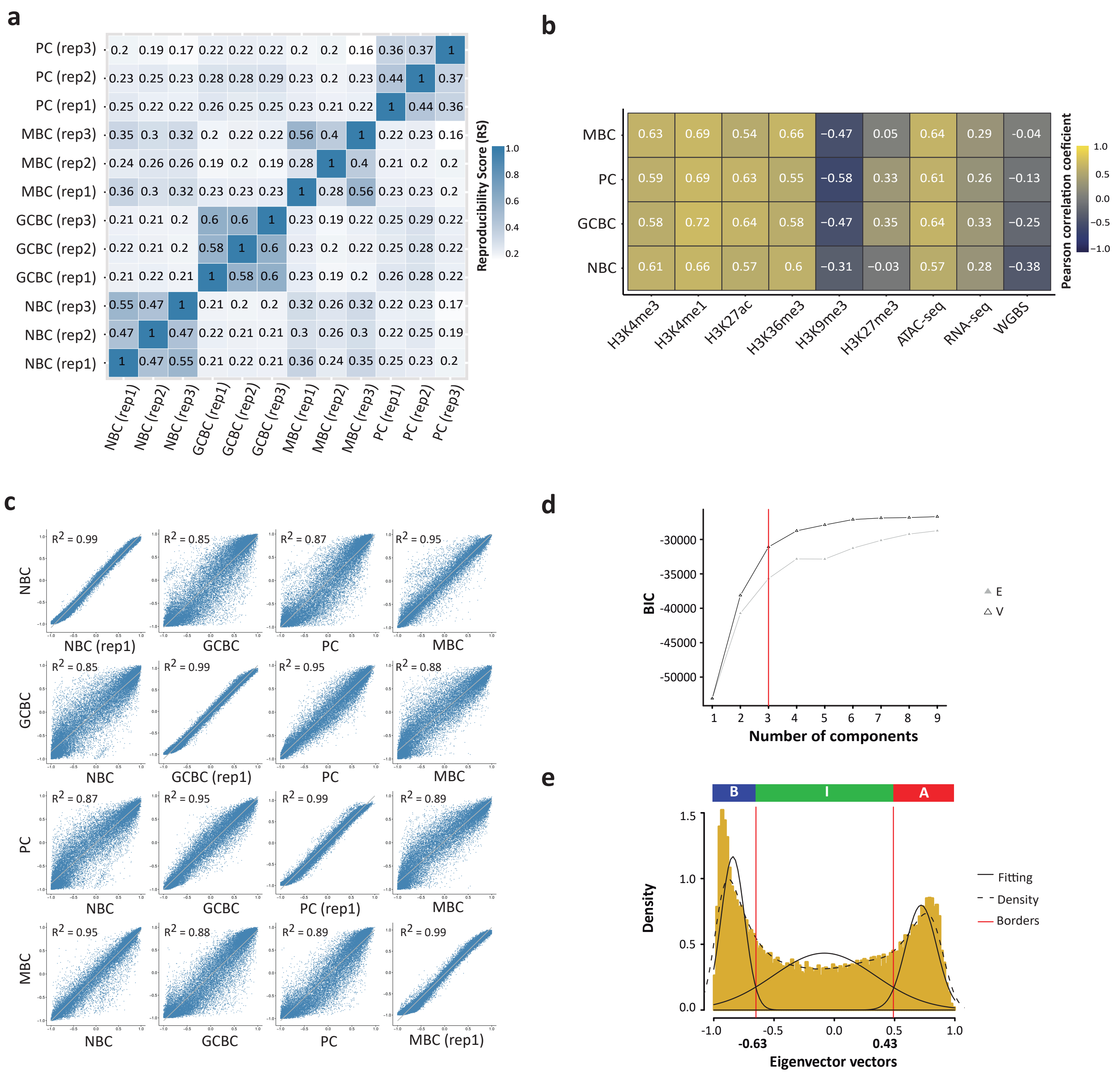

Supplementary Fig. 1. a Average genome-wide reproducibility score matrix of each B cell subpopulation replicate at 100 kb resolution. The reproducibility score ranging between 0 (totally different matrices) and 1 (identical matrices). **b** Pearson correlation between the eigenvector coefficients with six histone marks, chromatin accessibility (ATAC-seq), gene expression (RNA-seq) and DNA methylation (WGBS). Positive values of the eigenvector show higher correlation with H3K4me1 (enhancer mark) and chromatin accessibility. **c** Genome-wide scatterplots of coefficients from the first eigenvectors between pairs of B cell subpopulations at 100 kb resolution. The coefficient of determination (R^2) is indicated. **d** Bayesian Information Criterion (BIC) plot for the equal (E) and unequal (V) variance model parameterization ranged from 1 to 10 clusters. **e** Compartment definition model. The *x*-axis shows the distribution of the eigenvector coefficients and the *y*-axis indicates the density. The fitting model proposed is highlighted using solid black lines. The red lines mark the intersection points (IV1 = -0.63 and IV2 = +0.43) used to distinguish the three different compartments (A-type, I-type, B-type). Sample sizes were for NBC, GCBC, PC and MBC: *n* = 3 merged biologically independent samples.

Supplementary Fig. 2

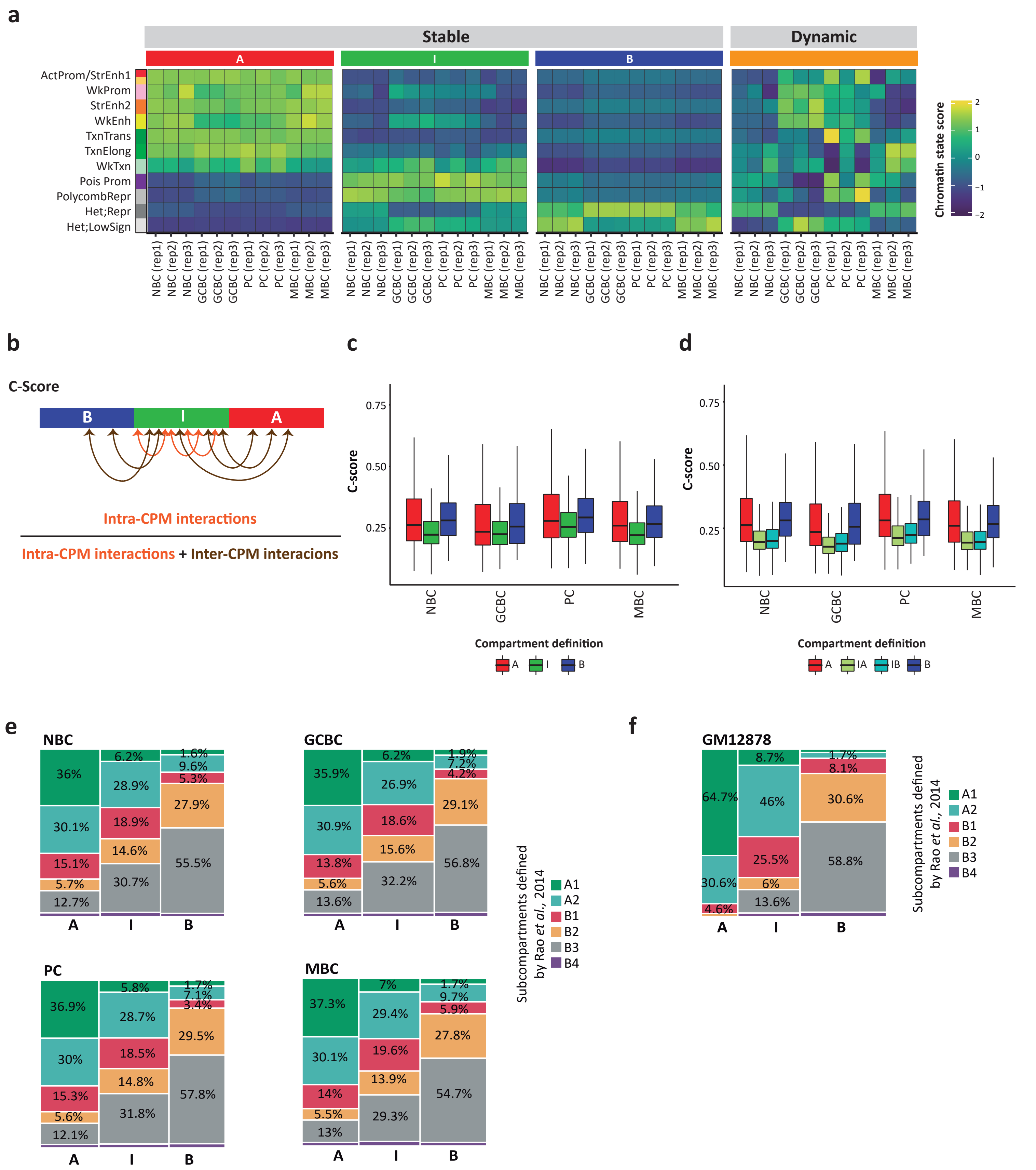

Supplementary Fig. 2. **a** Functional validation of the stable (A-type, I-type and B-type) and dynamic compartments in all B cell subpopulation replicates using eleven different chromatin states. The chromatin state score is normalized by sample and chromatin state. **b** C-score, a method defined by the ratio of contacts between regions within the same compartment (intra-compartment contacts) over the total chromosomal contacts per compartments (intra- and inter-chromosomal interactions). **c/d** C-score distributions at the three defined compartments A-type, I-type and B-type (**c**) and segmenting the I-type compartment onto positive (IA) or negative (IB) eigenvector coefficients (**d**). **e/f** Correlation between the three compartment segmentation and the six subcompartments defined by Rao et al.[12] in normal B cell subpopulations (**e**) and GM12878 (**f**). The percentage (>1%) of each subcompartment is indicated. Sample sizes were for NBC, GCBC, PC and MBC: $n = 3$ merged biologically independent samples. For the boxplots, centerline, box limits and whiskers represent the median, 25th and 75th percentiles and 1.5x interquartile range, respectively.

Supplementary Fig. 3

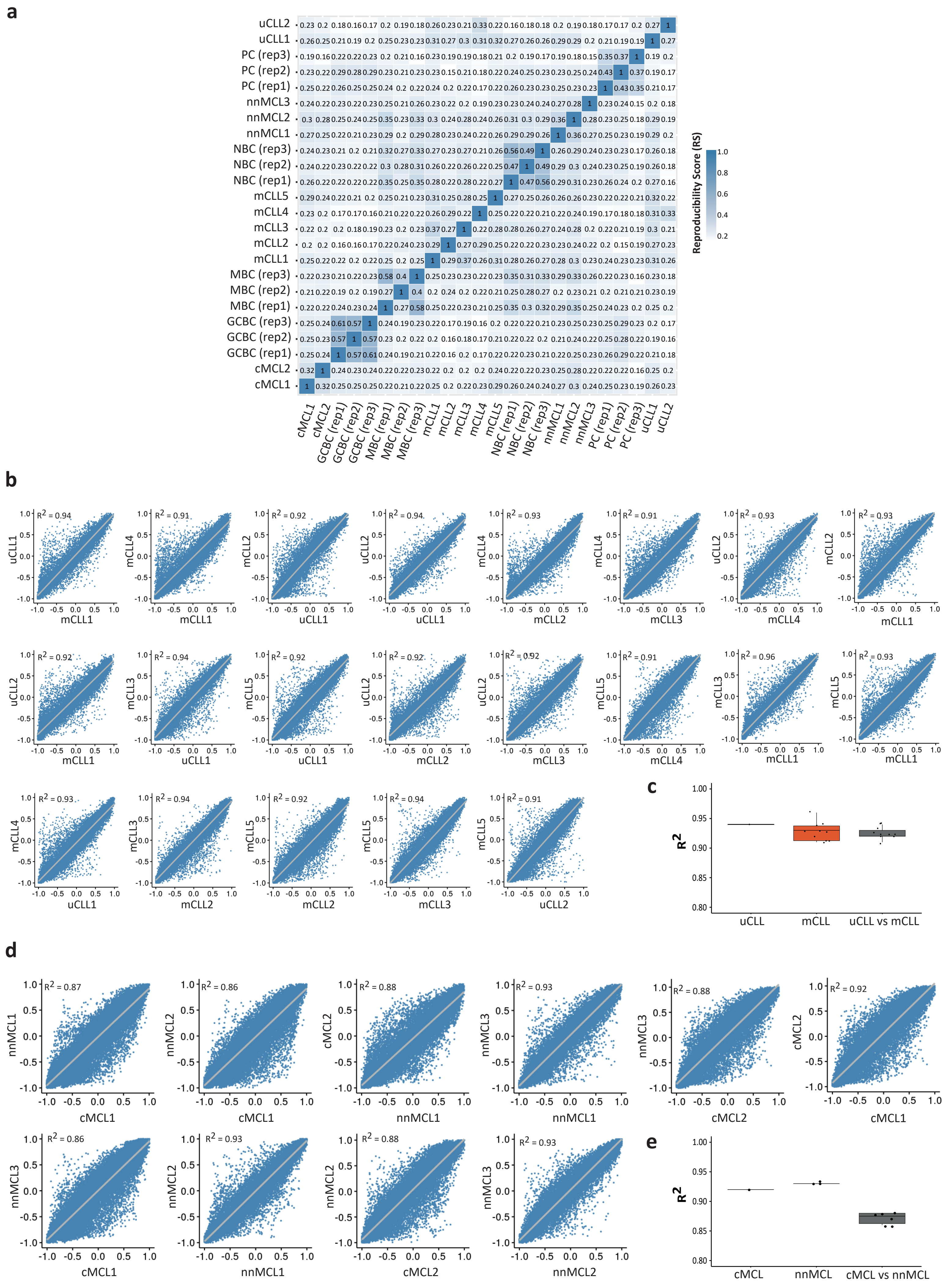

Supplementary Fig. 3. a Average genome-wide reproducibility score matrix of each B cell subpopulation replicate and B cell neoplasia at 100 kb. The reproducibility score ranging between 0 (totally different matrices) and 1 (identical matrices). **b/d** Genome-wide scatterplots of the first eigenvector showing the correlation between pairs of each B cell malignancy samples at 100 kb resolution. CLL cases are shown in panel **b** and MCL cases in panel **d**. The coefficient of determination (R^2) is indicated. **c/e** Boxplot of the coefficients of determination calculated intra- or inter- each neoplasia subtype. CLL cases are shown in panel **c** and MCL cases in panel **e**. Samples sizes were for uCLL: n = 2 biologically independent samples, for mCLL: n = 5 biologically independent samples, for cMCL: n = 2 biologically independent samples and for nnMCL: n = 3 biologically independent samples. For the boxplots, centerline, box limits and whiskers represent the median, 25th and 75th percentiles and 1.5x interquartile range, respectively.

Supplementary Fig. 4

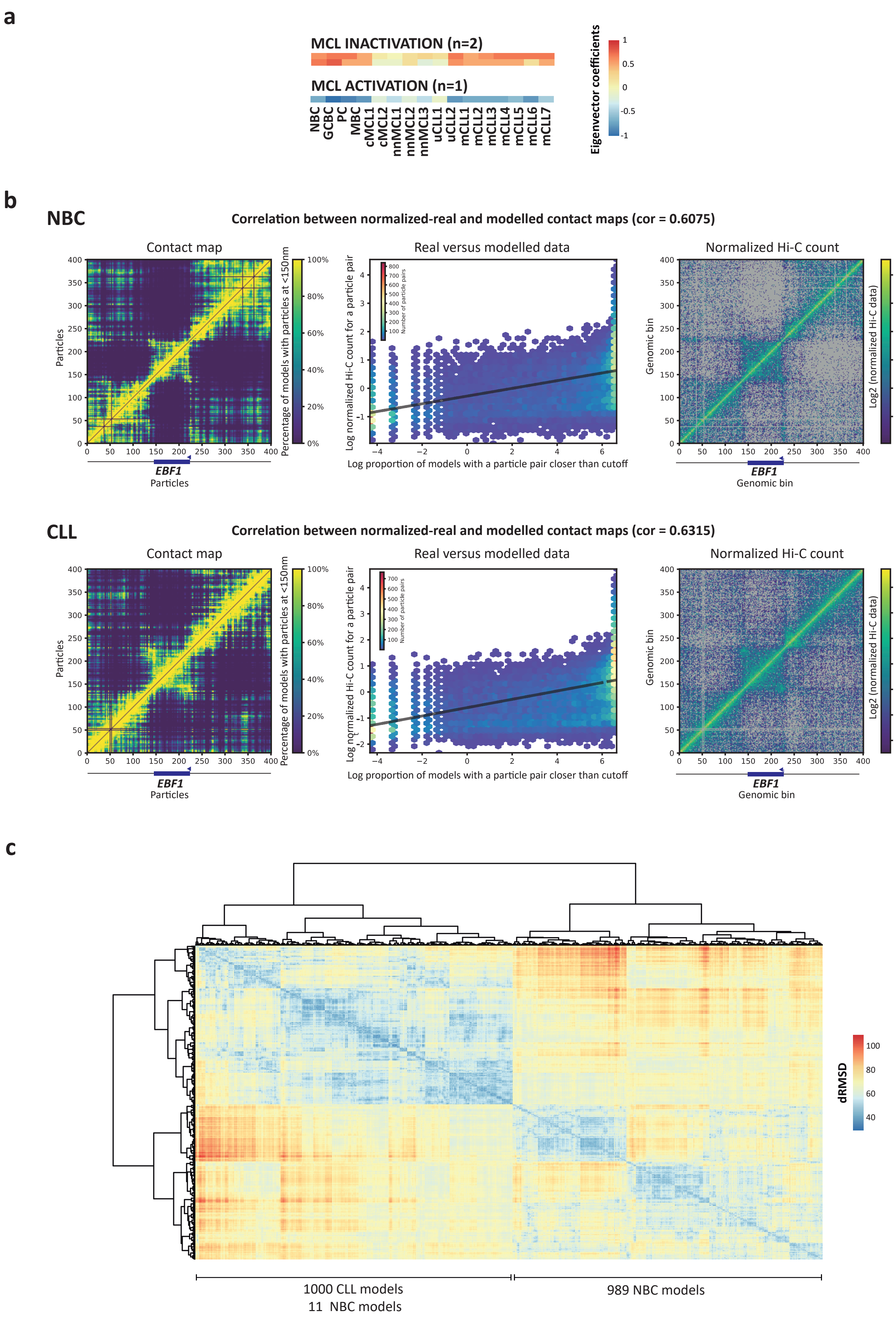

Supplementary Fig. 4. a Heatmap showing the eigenvector coefficients of the MCL specific compartments. *Top:* Losing activation. *Bottom:* Gaining activation. **b** Correlation between normalized Hi-C and modeled contact maps in *EBF1* regulatory landscape. *Top:* NBC. *Bottom:* CLL. *Left:* Contact map computed from the restraint-based model. *Middle:* Scatterplot of Hi-C normalized map vs. modeled contact data with linear regression. *Right:* Normalized Hi-C data. The position of *EBF1* is indicated in blue at the bottom of the matrix plots. **c** Heatmap of the hierarchical clustering of the dRMSD values computed for all the possible pairs of generated models (1,000 in NBC and 1,000 in CLL).

Supplementary Fig. 5

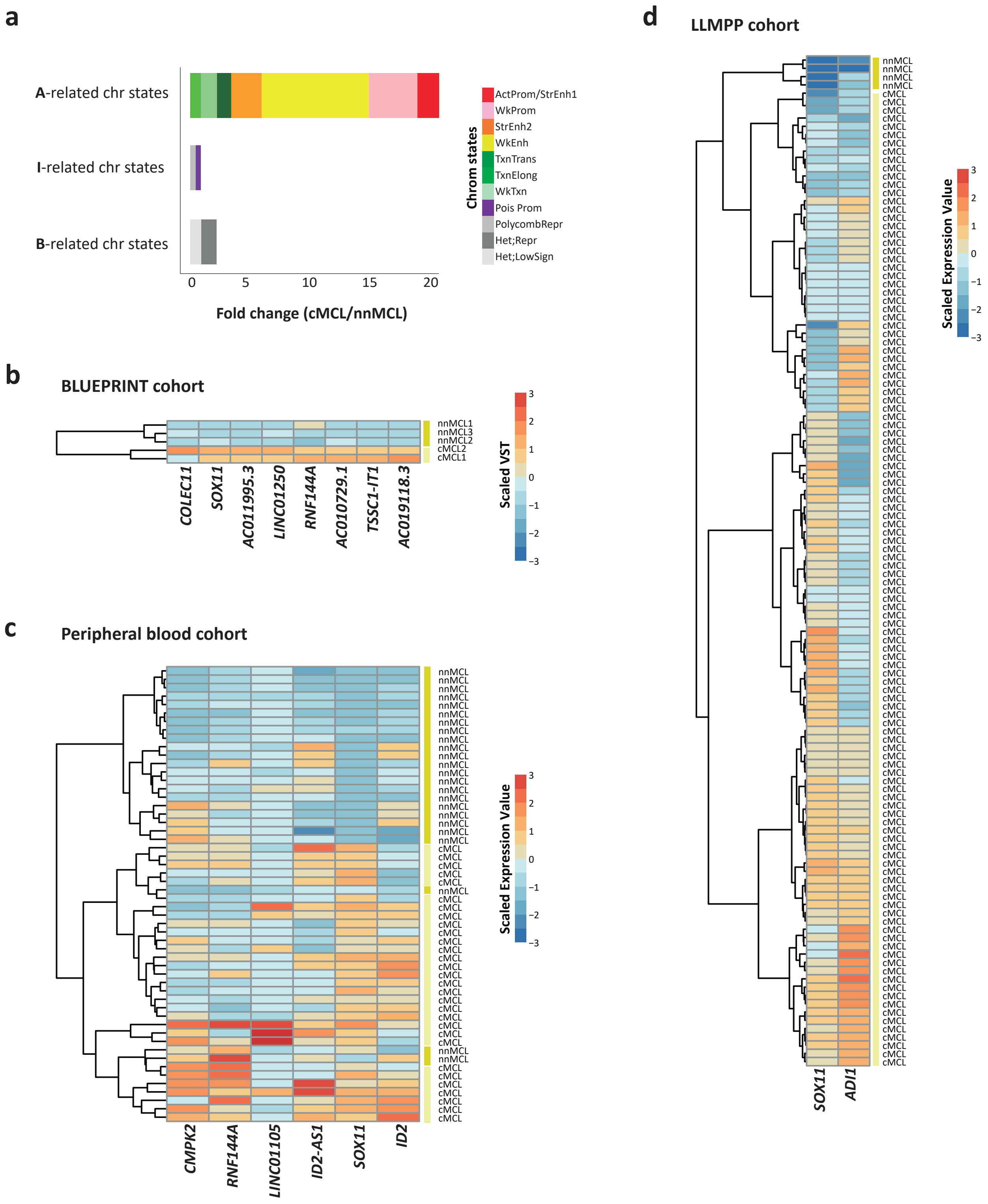

Supplementary Fig. 5. a Bar graphs represent the fold change between cMCL and nnMCL of each three groups of chromatin states (arranged by their relationship to the A-type, I-type and B-type compartments). **b-d** Heatmaps of the overexpressed genes located within 6.1 Mb region in each cohort: BLUEPRINT (FDR < 0.05) (**b**), peripheral blood (adjusted *p* value < 0.1) (**c**) and/or LLMPP (adjusted *p* value < 0.1) (**d**), with MCL samples classified as cMCL (light yellow) and nnMCL (dark yellow) subtypes. The VST and expression values were scaled by genes. ActProm-StrEnh1 active promoter-strong enhancer 1, WkProm weak promoter, StrEnh2 strong enhancer 2, WkEnh weak enhancer, TxnTrans transcription transition, TxnElong transcription elongation, WkTxn weak transcription, PoisProm poised promoter, PolycombRepr polycomb-repressed, Het;Repr heterochromatin-repressed, Het;LowSign heterochromatin-low signal.
