# Supplementary material for: Dynamics of genome architecture and chromatin function during human B cell differentiation and neoplastic transformation
Source: Nat Commun. 2021 Jan 28;12:651. doi: 10.1038/s41467-020-20849-y (PMC7844026; doi:10.1038/s41467-020-20849-y)
Supplement: Supplementary file 3 — Description of Additional Supplementary Files [file 41467_2020_20849_MOESM3_ESM.pdf]

Supplementary Data

Supplementary Data 1. In situ Hi-C experimental quality metrics.

Supplementary Data 2. GCBC specific 3D active compartments on a three-column bed file format (chromosome, start position and end position).

Supplementary Data 3. List of the identified enriched binding motifs expressed in GCBC. *P* values were calculated using the Wilcoxon rank-sum test (one-sided).

Supplementary Data 4. Genes differentially upregulated (FDR < 0.05 controlled by Benjamini and Hochberg test) in GCBC specific regions. The coordinates of the compartment or compartments hosting each gene are indicated.

Supplementary Data 5. Patient characteristics and general overview of the omics layers analyzed.

Supplementary Data 6. Genes differentially expressed (FDR < 0.05 controlled by Benjamini and Hochberg test) at CLL-specific inactive compartments. The coordinates of the compartment or compartments each gene belongs to are indicated.

Supplementary Data 7. Genes differentially expressed (FDR < 0.05 controlled by Benjamini and Hochberg test) at CLL-specific active compartments. The coordinates of the compartment or compartments each gene belongs to are indicated.

Supplementary Data 8. List of the identified enriched binding motifs expressed in CLL-specific active compartments. *P* values were calculated using the Wilcoxon rank-sum test (one-sided).
