# Supplementary material for: Dynamics of genome architecture and chromatin function during human B cell differentiation and neoplastic transformation
Source: Nat Commun. 2021 Jan 28;12:651. doi: 10.1038/s41467-020-20849-y (PMC7844026; doi:10.1038/s41467-020-20849-y)
Supplement: Supplementary file 12 — Reporting Summary [file 41467_2020_20849_MOESM12_ESM.pdf]

nature research

Corresponding author(s): M.A. Marti Renom
J.I. Martin-Subero

Last updated by author(s): Oct 21, 2020

Reporting Summary

Nature Research wishes to improve the reproducibility of the work that we publish. This form provides structure for consistency and transparency in reporting. For further information on Nature Research policies, see Authors & Referees and the Editorial Policy Checklist .

Statistics

For all statistical analyses, confirm that the following items are present in the figure legend, table legend, main text, or Methods section.

n/a	Confirmed	
☐	☒	The exact sample size (*n*) for each experimental group/condition, given as a discrete number and unit of measurement
☐	☒	A statement on whether measurements were taken from distinct samples or whether the same sample was measured repeatedly
☐	☒	The statistical test(s) used AND whether they are one- or two-sided<br>*Only common tests should be described solely by name; describe more complex techniques in the Methods section.*
☐	☒	A description of all covariates tested
☐	☒	A description of any assumptions or corrections, such as tests of normality and adjustment for multiple comparisons
☐	☒	A full description of the statistical parameters including central tendency (e.g. means) or other basic estimates (e.g. regression coefficient) AND variation (e.g. standard deviation) or associated estimates of uncertainty (e.g. confidence intervals)
☐	☒	For null hypothesis testing, the test statistic (e.g. *F*, *t*, *r*) with confidence intervals, effect sizes, degrees of freedom and *P* value noted<br>*Give P values as exact values whenever suitable.*
☐	☒	For Bayesian analysis, information on the choice of priors and Markov chain Monte Carlo settings
☒	☐	For hierarchical and complex designs, identification of the appropriate level for tests and full reporting of outcomes
☐	☒	Estimates of effect sizes (e.g. Cohen's *d*, Pearson's *r*), indicating how they were calculated

Our web collection on statistics for biologists contains articles on many of the points above.

Software and code

Policy information about availability of computer code

Data collection	No software has been used for data collection.
Data analysis	Multiples R packages have been used for data analysis such as stats version 3.5.1, mixtools version 1.2.0, mclust version 5.4.6, data.table version 1.13.0, base version 3.5.1, DESeq2 version 1.28.0, GenomicRanges version 1.34.0.<br>The downstream analysis of the Hi-C data were performed using TADbit version 0.4.62, ONED version 0.0.0.9100, HOMER version 4.9.1 and Python libraries as scipy.spatial and scipyndimage version 1.2.1. ChIP-seq and ATAC-seq were analysed by bwa version 0.7.7, Picard version 2.8.1, SAMtools version 1.3.1, MACS2 version 2.0.10.20131216 and 2.1.1.20160309, BEDtools version 2.25.0, DESeq2 version 1.28.0 R package. RNA-seq was processed using GRAPE2 pipeline with STAR version 2.4.0j and RSEM version 1.2.21 softwares (adapted from the ENCODE Long RNA-Seq pipeline). WGBS data was processed using GEM version 3.0. The integration of the multi-omics data was done using BEDOPS software version 2.4.28. The analysis of motif enrichment was done using AME-MEME suite version 5.0.3, AME-FIMO suite version 4.12. The microarrays were normalized using frma version 1.38.0 R package and limma version 3.42.2 R package was used to identify their differentially expressed genes. Specific plots of the manuscript were generated using pheatmap version 1.0.12, alluvial version 0.1.2, karyoplote version 1.14.1 and PhantomPeakQualTools version 1.1.0.

For manuscripts utilizing custom algorithms or software that are central to the research but not yet described in published literature, software must be made available to editors/reviewers. We strongly encourage code deposition in a community repository (e.g. GitHub). See the Nature Research guidelines for submitting code & software for further information.

Data

Policy information about availability of data

All manuscripts must include a data availability statement. This statement should provide the following information, where applicable:
- Accession codes, unique identifiers, or web links for publicly available datasets
- A list of figures that have associated raw data
- A description of any restrictions on data availability

In situ Hi-C data generated within this study is in the process of being deposited in a public data repository. The remaining raw data analyzed in this study has been deposited at the European Genome-Phenome Archive (EGA, http://www.ebi.ac.uk/ega/), which is hosted at the European Bioinformatics Institute (EBI).We have not yet created a specific dataset unifying all the raw data of our study, but all epigenomic data normal B cells, CLL and MCL can be found under accession numbers EGAS00001000326 (ChIPseq), EGAS00001001596 (ATACseq), EGAS00001000418 (WGBS) and EGAS00001000327 (RNAseq). It is important to note here that the consent agreements signed by the participants in the BLUEPRINT project do not allow anonymous access to the raw data. If reviewers wish to access raw data, they will need to disclose their identity. To help reviewers to visualize the generated EBF1 models using TADkit and the multi omics data in a browser session we have created a website accompanying the manuscript: http://resources.idibaps.org/paper/dynamics-of-genome-architecture-and-chromatin-function-during-human-b-cell-differentiation-and-neoplastic-transformation, which can be anonymously accessed.

Field-specific reporting

Please select the one below that is the best fit for your research. If you are not sure, read the appropriate sections before making your selection.

- [x] Life sciences
- [ ] Behavioural & social sciences
- [ ] Ecological, evolutionary & environmental sciences

For a reference copy of the document with all sections, see nature.com/documents/nr-reporting-summary-flat.pdf

Life sciences study design

All studies must disclose on these points even when the disclosure is negative.

Sample size	Sample-size was not determined by calculations. Each B cell subpopulations was studied in triplicates to avoid any technical issue and each B cell neoplasm was studied with the number of patient samples we had available material to perform all the protocols and experiments.
Data exclusions	No data was excluded.
Replication	The reproducibility was achieved performing a minimum of triplicates per each B cell subpopulation studied.
Randomization	Randomization was not applied in our study as for instance the patient samples were categorized according to their tumour subtype and we did not had a clinical research involving treatments.
Blinding	Due to the experimental design and goals of our article sample blinding was not neccessary.

Reporting for specific materials, systems and methods

We require information from authors about some types of materials, experimental systems and methods used in many studies. Here, indicate whether each material, system or method listed is relevant to your study. If you are not sure if a list item applies to your research, read the appropriate section before selecting a response.

Materials & experimental systems

n/a	Involved in the study	
☐	☒	Antibodies
☒	☐	Eukaryotic cell lines
☒	☐	Palaeontology
☒	☐	Animals and other organisms
☐	☒	Human research participants
☒	☐	Clinical data

Methods

n/a	Involved in the study	
☐	☒	ChIP-seq
☒	☐	Flow cytometry
☒	☐	MRI-based neuroimaging

Antibodies

Antibodies used	Antibodies used are defined on the BLUEPRINT guidelines: http://www.blueprint-epigenome.eu/index.cfm?p=D6F8811F-DACF-7979-CEAC0B9034C28037. This website is a sublink of the website referred to in the manuscript where the ChIP-seq and ATAC-seq protocol can be found: http://www.blueprint-epigenome.eu/index.cfm?p=7BF8A4B6-F4FE-861A-2AD57A08D63D0B58.<br>Catalog numbers of antibodies (Diagenode) used are H3K27ac: C15410196/pAb-196-050 (LOT: A1723-0041D), H3K4me1:

	C15410194/pAb-194-050 (LOT: A1863-001P), H3K4me3: C15410003-50/pAb-003-050 (LOT: A5051-001P), H3K36me3: C15410192/(pAb-192-050 (LOT: A1847-001P), H3K9me3: C15410193/pAb-193-050 (LOT: A1671-001P), H3K27me3: C15410195/pAb-195-050 (LOT: A1811-001P).
Validation	Antibodies were validated as defined by the BLUEPRINT guidelines: http://www.blueprint-epigenome.eu/index.cfm?p=D6F8811F-DACF-7979-CEAC0B9034C28037. This website is a sublink of the website referred to in the manuscript where the ChIP-seq and ATAC-seq protocol can be found: http://www.blueprint-epigenome.eu/index.cfm?p=7BF8A4B6-F4FE-861A-2AD57A08D63D0B58.

Human research participants

Policy information about studies involving human research participants

Population characteristics	The studied cohort analysed in this study contained peripheral blood samples of 7 chronic lymphocytic leukemia (CLL) patients and 5 mantle cell lymphoma patients (Supplementary Table 5) as well as and mature B cells of healthy male donors, isolated from peripheral blood (from 56 to 61 years of age) or tonsils (from 2 to 12 years of age).
Recruitment	Patients were diagnosed and treated in the Hospital Clínic of Barcelona. For the present study, patients were selected based on the amount of available cryopreserved cells to perform all the multi-omics experiments, stored at the hematopathology collection of the Hospital Clínic. Mature B cells were obtained from either Banc de Sang i Teixits or Clínica Universidad de Navarra.
Ethics oversight	The enrolled patients or legally authorized representatives/parents of minor participants (age below 18 years of age),gave informed consent for scientific study following the ICGC guidelines and the ICGC Ethics and Policy committee. This study was approved by the clinical research ethics committee of the Hospital Clínic of Barcelona.

Note that full information on the approval of the study protocol must also be provided in the manuscript.

ChIP-seq

Data deposition

- [x] Confirm that both raw and final processed data have been deposited in a public database such as GEO.
- [x] Confirm that you have deposited or provided access to graph files (e.g. BED files) for the called peaks.

Data access links<br>*May remain private before publication.*	Raw data: http://www.ebi.ac.uk/ega accession number: EGAS00001000326.
Files in database submission	Files were submitted as part of the BLUEPRINT consortium under the accession number: EGAS00001000326.
Genome browser session<br>(e.g. UCSC)	A genome browser session of all the data presented in this study is available in the website accompanying the article: http://resources.idibaps.org/paper/dynamics-of-genome-architecture-and-chromatin-function-during-human-b-cell-differentiation-and-neoplastic-transformation

Methodology

Replicates	For all mature B-cell subpopulations 3 biological replicates were performed.
Sequencing depth	Aimed average sequencing depth for ChIP-seq experiments was around 30 million reads per sample.
Antibodies	Antibodies used are defined on the BLUEPRINT guidelines: http://www.blueprint-epigenome.eu/index.cfm?p=D6F8811F-DACF-7979-CEAC0B9034C28037. This website is a sublink of the website referred to in the manuscript where the ChIP-seq can be found: http://www.blueprint-epigenome.eu/index.cfm?p=7BF8A4B6-F4FE-861A-2AD57A08D63D0B58.<br>Catalog numbers of antibodies (Diagenode) used are H3K27ac: C15410196/pAb-196-050 (LOT: A1723-0041D), H3K4me1: C15410194/pAb-194-050 (LOT: A1863-001P), H3K4me3: C15410003-50/pAb-003-050 (LOT: A5051-001P), H3K36me3: C15410192/(pAb-192-050 (LOT: A1847-001P), H3K9me3: C15410193/pAb-193-050 (LOT: A1671-001P), H3K27me3: C15410195/pAb-195-050 (LOT: A1811-001P).
Peak calling parameters	Peaks were called as described in http://dcc.blueprint-epigenome.eu/#/md/methods using MACS2 (version 2.0.10.20131216) with input control.
Data quality	All assigned peaks had an FDR<0.05. The respective mean and median number of regions with fold enrichment above 5 per sample were for H3K4me1 82.2% and 82.1%; H3K4me3 98.8% and 98.9%; and H3K27ac 97.5% and 98.8%. For the broad histone modifications enrichment ratios tend to be lower in general, which is observed in other studies as well, for H3K36me3 7.9% and 6.5%; for H3K27me3 2.6% and 0.5%; and for H3K9me3 1.6% and 0.5%. A less stringent cutoff of fold enrichment of 2 (instead of 5) increases these numbers. The respective mean and median number of regions with fold enrichment above 2 per sample were of 100% for all histone marks analyzed.

Software

Fastq files of ChIP-seq data were aligned to the GRCh38 reference genome using bwa 0.7.7, PICARD (http://broadinstitute.github.io/picard/) and SAMTOOLS, and wiggle plots were generated (using PhantomPeakQualTools R package) as described http://dcc.blueprint-epigenome.eu/#/md/methods.
