# Supplementary material for: Dynamics of genome architecture and chromatin function during human B cell differentiation and neoplastic transformation
Source: Nat Commun. 2021 Jan 28;12:651. doi: 10.1038/s41467-020-20849-y (PMC7844026; doi:10.1038/s41467-020-20849-y)
Supplement: Supplementary file 2 — Peer Review File [file 41467_2020_20849_MOESM2_ESM.pdf]

Editorial Note: This manuscript has been previously reviewed at another journal that is not operating a transparent peer review scheme. This document only contains reviewer comments and rebuttal letters for versions considered at *Nature Communications*.

REVIEWERS' COMMENTS

Reviewer #1 (Remarks to the Author):

The manuscript has improved. It is an important resource for the B cell community and beyond. It is suitable for publication.

Reviewer #2 (Remarks to the Author):

the authors have answered for the most part my questions

Response to Reviewers:

Comments from reviewers

No additional comments were raised by reviewers
